# Gradient of Segmental Dynamics in Stereoregular Poly(methyl methacrylate) Melts Confined between Pristine or Oxidized Graphene Sheets

**DOI:** 10.3390/polym13050830

**Published:** 2021-03-08

**Authors:** Alireza Foroozani Behbahani, Vagelis Harmandaris

**Affiliations:** 1 Institute of Applied and Computational Mathematics, Foundation for Research and Technology-Hellas, GR-71110 Heraklion, Greece; 2 Department of Mathematics and Applied Mathematics, University of Crete, GR-70013 Heraklion, Greece; 3 Computation-Based Science and Technology Research Center, The Cyprus Institute, 2121 Nicosia, Cyprus

**Keywords:** segmental dynamics, α-relaxation, confinement, interfaces, poly(methyl methacrylate), tacticity, graphene, graphene oxide, surface–chemistry, simulations

## Abstract

Segmental dynamics in unentangled isotactic, syndiotactic, and atactic poly(methyl methacrylate) (i-, a-, and s-PMMA) melts confined between pristine graphene, reduced graphene oxide, RGO, or graphene oxide, GO, sheets is studied at various temperatures, well above glass transition temperature, via atomistic molecular dynamics simulations. The model RGO and GO sheets have different degrees of oxidization. The segmental dynamics is studied through the analysis of backbone torsional motions. In the vicinity of the model nanosheets (distances less than ≈2 nm), the dynamics slows down; the effect becomes significantly stronger with increasing the concentration of the surface functional groups, and hence increasing polymer/surface specific interactions. Upon decreasing temperature, the ratios of the interfacial segmental relaxation times to the respective bulk relaxation times increase, revealing the stronger temperature dependence of the interfacial segmental dynamics relative to the bulk dynamics. This heterogeneity in temperature dependence leads to the shortcoming of the time-temperature superposition principle for describing the segmental dynamics of the model confined melts. The alteration of the segmental dynamics at different distances, *d*, from the surfaces is described by a temperature shift, $\Delta T_{\mathrm{seg}}\left(d\right)$ (roughly speaking, shift of a characteristic temperature). Next, to a given nanosheet, i-PMMA has a larger value of $\Delta T_{\mathrm{seg}}$ than a-PMMA and s-PMMA. This trend correlates with the better interfacial packing and longer trains of i-PMMA chains. The backbone torsional autocorrelation functions are shown in the frequency domain and are qualitatively compared to the experimental dielectric loss spectra for the segmental α -relaxation in polymer nanocomposites. The $\varepsilon_{T}^{{}^{\prime \prime }}\left(f\right)$ (analogous of dielectric loss, $\varepsilon^{{}^{\prime \prime }}\left(f\right)$ , for torsional motion) curves of the model confined melts are broader (toward lower frequencies) and have lower amplitudes relative to the corresponding bulk curves; however, the peak frequencies of the $\varepsilon_{T}^{{}^{\prime \prime }}\left(f\right)$ curves are only slightly affected.

## 1. Introduction

Interfaces alter the dynamics of the neighboring polymer chains. Understanding interfacial polymer dynamics is important in relation to a variety of polymer materials, including nanocomposites and thin films [1,2]. Due to these practical applications and also basic scientific motivations, for example, regarding understanding the nature of glass transition temperature (*T_g_*), the dynamics of interfacial polymer systems have been extensively studied. However, providing a detailed description for the interfacial dynamics and assessing the effects of different parameters on it are still active areas of research.

Interfacial polymer dynamics has been extensively studied experimentally through various techniques. For example, Papon et al. [3] studied the segmental relaxation of poly(ethyl acrylate) in the presence of silica nanoparticles using nuclear magnetic resonance (NMR) and differential scanning calorimetry (DSC) experiments. They described the results of both NMR and DSC measurements in terms of a surface induced gradient of local *T_g_*; the local *T_g_*, was found to be the maximum at the nanoparticle surface and converges to the bulk value after few nanometers. Wei and Torkelson [4] inserted dye molecules at different locations in the nanocomposites of poly(2-vinyl pyridine) and silica and used fluorescence technique to study *T_g_* at those locations; increases of *T_g_* at the silica interface were reported. Choi et al. [5] used neutron reflectivity to measure the diffusion coefficients of PMMA and polystyrene melts confined between graphene oxide sheets; for highly confined PMMA films (film thickness less than ≈3 bulk gyration radius), large decreases of diffusion coefficient were reported. Dielectric relaxation spectroscopy experiments have also been extensively used to study the interfacial polymer dynamics, particularly the interfacial segmental dynamics. Quantities like the thickness of the interfacial polymer layer, the relaxation times, and local *T_g_* at the interfacial layer, and the temperature dependence of the interfacial dynamics have been studied by dielectric relaxation spectroscopy measurements [6,7,8,9,10,11,12,13,14,15,16]. The results of dielectric spectroscopy experiments are discussed in more detail in the main text and are compared to the simulation results.

To study the interfacial properties of polymers, molecular simulation techniques are rewarding. The accessible time scales of atomistic simulations are far from the experimental time scales; however, simulations can provide direct and high resolution data about the behavior of well-defined interfacial systems. Few representative works concerning the simulation of the dynamics of chemically realistic (atomistic) models of interfacial polymer systems (mainly containing carbon-based solid surfaces) include the following: Harmandaris et al. [17] studied the segmental and chain dynamics in a thin film of polyethylene adsorbed on graphite. Paul and coworkers [18,19,20] analyzed the dynamics of 1-4-Polybutadiene confined between graphite walls using molecular dynamics simulations. They discussed the slow process of mass exchange between layers close to the walls and analyzed dielectric relaxation, segmental reorientation, and local incoherent intermediate scattering function near the confining surfaces. Pandey et al. [21] used a multiscale simulation methodology to study the local dynamics and conformational properties of polyisoprene adsorbed on graphite. The deceleration of local orientational dynamics close to graphite was observed. Furthermore, they observed that longer trains (consecutively adsorbed monomers of a chain) have slower local dynamics than the shorter ones. Vogiatzis and Theodorou [22] studied local segmental dynamics and stresses in molten polystyrene/C ${}_{60}$ nanocomposites. Bačová et al. [23] studied the dynamics of polyethylene and poly(ethylene oxide) in the nanocomposites of edge functionalized (hydrogenated and carboxylated) graphene sheets. Because of the polar interactions, a big retardation of dynamics is observed for poly(ethylene oxide) near the carboxyl functional groups. Kritikos et al. [24] studied the nanocomposites of polystyrene and poly(acrylic acid) with pristine graphene and graphene oxide. The decelerations of torsional and orientational dynamics were reported for both polymers close to the two nanosheets. Recently, Mapesa et al. [16] studied the segmental dynamics in thin films of poly(vinyl acetate) casted on a silica substrate using molecular dynamics simulations combined with dielectric relaxation spectroscopy. Faster segmental relaxation close to the free surface and slower relaxation close to the attractive silica surface have been reported.

Graphene-based nanosheets are promising candidates for applications in polymer nanocomposites. The graphene-based nanosheets that are used in polymer nanocomposites usually contain functional groups. Particularly, oxidized graphene nanosheets are widely used [25,26]. The exfoliation of graphite oxide in polar solvents is a scalable solution-based process for the synthesis of graphene-based nanosheets; this process leads to the production of graphene oxide (GO) which is a graphene-based sheet containing a large concentration of functional groups like hydroxyl, epoxide, and carboxyl [25,26,27,28,29,30]. Through thermal or chemical reduction, GO is reduced to a nearly graphene-like conjugated nanosheets, which is called reduced graphene oxide (RGO). Usually, the reduction is not complete and RGO has some remaining functional groups.

Poly(methyl methacrylate) (PMMA) is a disubstituted vinyl polymer whose bulk properties depend on its tacticity. For example, isotactic PMMA (i-PMMA) has a lower *T_g_* and a higher characteristic ratio ( $C_{\infty }$ ) than syndiotactic PMMA (s-PMMA) [31,32]. The *T_g_*s of high molecular weight i-PMMA and s-PMMA are around 320 K and 400 K, respectively [31]. Furthermore, there are some experimental reports about the tacticity dependent interfacial properties of PMMA. For example, using ellipsometry technique, for thin films of i-PMMA casted on SiO ${}_{2}$ and Al ${}_{2}$ O ${}_{3}$ substrates, an increase of *T_g_* and for thin film of s-PMMA casted on the same substrates, a decrease of *T_g_* relative to the bulk value, have been reported [33]. Similarly, for solvent blended nanocomposites of i-PMMA containing PG or RGO, an increase of *T_g_* relative to the bulk value has been reported, whereas, for nanocomposites of atactic (syndiotactic-rich) PMMA, no shift has been detected [34]. Besides possessing tacticity-dependent properties, PMMA is a polar polymer that can interact with the functional groups of RGO and GO. Particularity, the carbonyl oxygen atoms of PMMA form hydrogen bonds with hydroxyl groups of RGO and GO.

Recently, we studied the interfacial structure and conformation of i-, a-, s-PMMA chains close to pristine graphene (PG), RGO, and GO [35]. Here, we extend the previous work and investigate the interfacial segmental dynamical properties of these systems at different temperatures (well above *T_g_*). The model nanosheets have practical importance in the synthesis of polymer nanocomposites; furthermore, they allow for the study of the effects of surface roughness and polymer/surface interactions on the interfacial polymer dynamics. In addition, PMMA chains form tacticity-dependent interfacial structures [35], and investigating the segmental dynamics of PMMA chains of different tacticities allows for an investigation of the correlation between interfacial structure and segmental dynamics. In the current work, the interfacial segmental dynamics are studied in detail at various temperatures. The surface induced gradient of the segmental dynamics and the average dynamics of the entire confined melts are discussed. The alteration of the segmental dynamics at different distances from the confining surfaces is described by a temperature shift (roughly speaking, shift of a characteristic temperature). Such a description provides an intuitive picture about the effects of confining surfaces on the segmental dynamics and has not often been provided in previous simulation works. Particular attention is paid to qualitatively comparing the simulation results to the available experimental dielectric spectra of the segmental α -relaxation in polymer nanocomposites and thin film and to provide some insights related to dielectric spectroscopy experiments.

The paper is organized as follows: In Section 2, the model systems and the simulation procedure are described. In Section 3, the results about the interfacial structure of stereoregular PMMA chains are reviewed. The interfacial gradient of segmental dynamics is discussed in Section 4.1 and Section 4.2. In Section 4.3, the overall dynamics of the confined melts are discussed. In Section 4.4, the segmental relaxation curves are shown in the frequency domain and are compared to the experimental dielectric α -relaxation spectra for polymer nanocomposites. Finally, in Section 5, a summary of the results is presented.

## 2. Model and Method

The RGO and GO model nanosheets were generated based on the Lerf–Klinowski structural model of graphite oxide [29,30]. Based on this model, GO contains unoxidized graphitic islands which are separated by 6-membered oxidized rings containing hydroxyl and epoxide groups and carbon double bonds [29,30]. There are also carboxyl groups on the edges of oxidized graphene flakes [30]; however, to model large scale GO and RGO sheets, we consider periodic nanosheets and there are no edge groups. In addition, the structural defects (e.g., holes) have not been included in the GO and RGO model structures. The difference between the model GO and RGO nanosheets is their degrees of oxidation expressed in terms of carbon-to-oxygen (C/O) ratio. For GO, C/O ratio is 3, and, for RGO, it is 10; these values correspond to typical experimental values of the C/O ratio for GO and RGO [25,27,28,34]. For both RGO and GO, concentrations of epoxied groups and hydroxyl groups are equal. The details of the generation of the RGO and GO model structures are provided elsewhere [35]. To describe PG and sp ${}^{2}$ carbon atoms of GO and RGO, a modified Dreiding force field has been used [21,36,37]. This force field describes the equilibrium spacing between graphene sheets in graphite [38] and the geometry and phonon structure of graphite and fullerene [37,38,39]. For oxidized regions of GO and RGO, the Dreiding force field has been used [40]. Since the Dreiding force field does not have partial atomic charges, the partial charges of the epoxide and hydroxyl groups were assigned based on the reported [41] charges for ethers and alcohols, respectively. The force field parameters for pristine and oxidized graphene sheets are provided in Table S1 (of Supplementary Materials).

For PMMA, an all atom force field has been employed that fairly describes the bulk melts of stereoregular PMMA [42,43,44]. The PMMA force field is provided in Table S2. Both PMMA and graphene force fields exclude non-bonded interactions between the first and second chemically bonded neighbors. The Lorentz–Berthelot combination rule was used for calculating the Van der Waals interactions between dissimilar atoms (including interactions between PMMA and the model graphene-based nanosheets).

**Figure 1:**
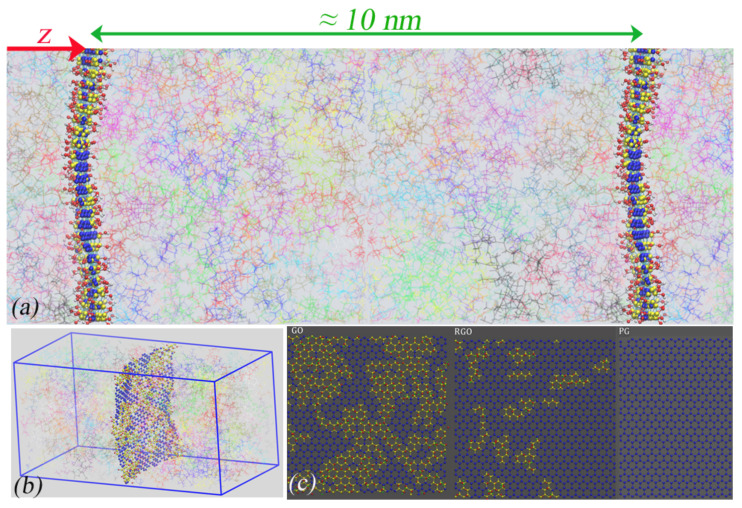
(**a**) The studied model geometry. A top view snapshot of the simulation box and one of its periodic images is presented. PMMA chains are shown by thin transparent lines. (**b**) A 3D snapshot of the simulation box containing one periodic GO nanosheet. (**c**) Snapshots of the model PG, RGO, and GO nanosheets. Graphitic and oxidized carbon atoms of GO are shown by blue and yellow colors, respectively. Further representative snapshots have been provided elsewhere [35].

Atomistic molecular dynamics (MD) simulations have been carried out on unentangled stereoregular (i-PMMA, s-PMMA) and atactic (a-PMMA) melts confined between PG, RGO, or GO surfaces. The PMMA chains contain 20 chemical repeat units ( $R_{g}$ ≈ 1 nm). The simulation box dimensions are approximately 5×5×10 nm ${}^{3}$ along *x*, *y*, and *z* directions, respectively. A single, periodic, and flexible PG, RGO, or GO sheet was placed in the simulation box, parallel to the xy plane, and periodic boundary conditions were applied along all directions. The periodic boundary conditions make the model geometry as a multi-layered film containing alternating ≈10 nm thick layers of PMMA confined between infinite sheets. The degree of the confinement of the model chains in the model geometry is rather moderate: $D/(2R_{g})\approx 5$ , where *D* is the film thickness. The numbers of basal carbon atoms of the model PG, RGO, and GO sheets are equal; however, because of the presence of the functional groups, RGO and GO are heavier than PG. The weight fractions of PG, RGO, and GO in the model systems are 6.98 , 7.86 , and 9.87 wt %, respectively; also their estimated volume fractions are 3.92 , 4.24 , and 5.22 vol %, respectively. Snapshots of the simulation box and the model nanosheets are provided in Figure 1.

The simulations were conducted using the open source package GROMACS [45]. The model PMMA stereoisomers (confined between PG, RGO, or GO) have been studied at three different temperatures, 580 K, 550 K, and 520 K, whereas a-PMMA/RGO and a-PMMA/GO systems were simulated at 580 K. As reference systems, the corresponding bulk PMMA melts were also simulated at 580 K, 550 K, 520 K, and 490 K. These temperatures are well above the bulk *T_g_* of the model PMMA chains (≈ T> *T_g_* +100 K) [31,44]. Performing the simulations at temperatures well above *T_g_* is a consequence of the computational limitations of the atomistic simulations. At T= *T_g_* the segmental relaxation time of polymers is around 100 s [9], whereas the accessible time scales of the atomistic simulations are limited to around 1 μ s–10 μ s; thus, the simulation temperature should be increased to make the segmental relaxation time within the accessible range. Therefore, the atomistic simulation of high *T_g_* polymers (e.g., PMMA or polystyrene) are often performed at temperatures higher than the usual experimental temperatures. In the current work, the results were mainly collected at 520 K–580 K. We should also state here that the thermal decomposition of PMMA chains, which might occur for experimental samples at high temperatures, do not happen in our model PMMA systems because chemical bonds are stable. Indeed, in the current model, chemical bonds are described, as in most atomistic models, via harmonic potentials that do not allow bond breaking. In cases in which such phenomena are important, e.g., for investigation of thermal degradation, reactive force fields can be used. For each system at each temperature, an equilibration run (around 150 ns–650 ns, depending on temperature and tacticity) was conducted. The equilibrated configurations at higher temperatures were used as the initial configurations for the lower temperatures. The length of the production runs were from around 0.25μ s up to 1.0μ s. More details about the model and simulation procedure are reported in the previous work [35].

## 3. Structural Properties

**Figure 2:**
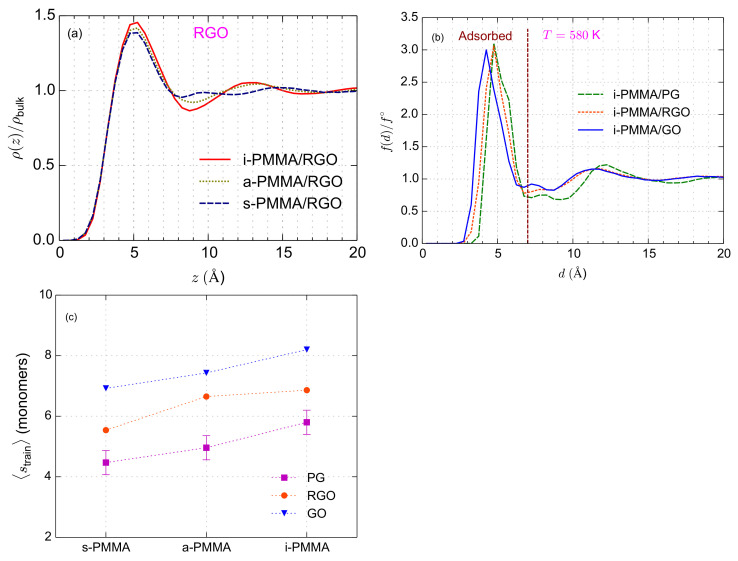
(**a**) Interfacial mass density profiles for PMMA stereoisomers near RGO. In this panel, z=0 corresponds to the center-of-mass of RGO. (**b**) Distribution of (minimum) distance between monomer centers-of-mass and nanosheet atoms for i-PMMA close to the model surfaces. (**c**) Weighted average number of monomers that belong to a train (or weighted average size of trains) for PMMA stereoisomers adsorbed on the model surfaces. The data are calculated at T=580 K. More details are provided in a previous work [35].

The structural properties of the model interfacial systems have been extensively discussed elsewhere [35]. Here, we only review some of the key results. Figure 2a shows, for the PMMA/RGO systems, the interfacial atomic mass density profiles of PMMA stereoisomers as a function of axial distance, *z*, from the center-of-mass of the RGO nanosheet. The density profiles are normalized with the respective bulk densities. For all confined PMMA systems (i-, a-, and s-PMMA melts), density profile peaks near the RGO surface (at a distance of around 5 Å) are observed, showing layered-like structures of PMMA atoms close to the surface. However, the density profile peak is higher and has a larger surface area for i-PMMA; furthermore, a small secondary peak is seen for i-PMMA at distances of around 12 Å from the RGO surface. These differences between density profiles suggest a better interfacial packing of i-PMMA than a-PMMA and s-PMMA. Similar behaviors have also been observed for PMMA/PG and PMMA/GO systems [35].

With increasing the concentration of functional groups, because of the excluded volume interactions and alteration of the hybridization of the oxidized carbon atoms from sp ${}^{2}$ to sp ${}^{3}$ , the roughness and curvature of graphene-based nanosheets increase. Because of different degrees of roughness and curvature, instead of the interfacial density profile, we probe the distribution of monomer/surface minimum-distance, f(d) , for assessing surface–chemistry dependence of the interfacial packing of PMMA. For the calculation of f(d) , the minimum distance between each monomer center-of-mass and the surface atoms (all of them, including functional group atoms) is considered. The f(d) curves are presented in a normalized form, $f\left(d\right)/f^{\circ }$ , where $f^{\circ }$ is the value of *f* far from the surface. The plots of $f\left(d\right)/f^{\circ }$ for i-PMMA near PG, RGO, and GO surfaces are shown in Figure 2b. Near all model surfaces, $f\left(d\right)/f^{\circ }$ exhibits a large peak indicating the organization of an adsorbed layer of i-PMMA monomers. The height of this peak is almost similar for the model surfaces. However, because of the roughness of the RGO and GO sheets and the preference of PMMA carbonyl groups to come close to the surface hydroxyl groups [35], the peak appears at smaller distances to the RGO and GO sheets than to the PG one. For i-PMMA, a small peak is also observed after the large peak which shows the partial organization of a second layer of i-PMMA monomers close to the surfaces. The first peak of $f\left(d\right)/f^{\circ }$ extends up to around 7 Å from the surfaces. Based on the width of this peak, we consider a PMMA monomer as adsorbed on a surface, if the minimum distance between its center-of-mass and the surface atoms is less than 7 Å.

Adsorbed chains are defined as chains that have at least one adsorbed monomer. The monomers of an adsorbed chain can be categorized as belonging to: *trains* (consecutively adsorbed monomers of a chain), *loops* (monomers connecting two trains), *tails* (monomers between a train and a chain end). Here, we present data about the (weighted) average size of trains ( $\langle s_{\mathrm{train}}\rangle \;=\sum_{s}\;s^{2}N\left(s\right)/\sum_{s}sN\left(s\right)$ , where N(s) is the number of trains containing *s* monomers) in Figure 2c. $\langle s_{\mathrm{train}}\rangle$ is tacticity and surface–chemistry dependent; with increasing the concentration of isotactic sequences of PMMA as well as the concentration of surface functional groups, the average size of trains increases. The higher roughness of the functionalized sheets and the electrostatic interactions (hydrogen bonds) of their hydroxyl groups with the carbonyl oxygen atoms of PMMA can explain the surface–chemistry dependence of $\langle s_{\mathrm{train}}\rangle$ . The higher stiffness (longer Kuhn segment and higher characteristic ratio) of i-PMMA chains than a-PMMA and s-PMMA ones can also explain the tacticity dependence of $\langle s_{\mathrm{train}}\rangle$ (characteristic ratios of the model i-PMMA, a-PMMA, and s-PMMA oligomers are 7.6 , 6.9 , and 6.5 at 580 K). The better interfacial packing of i-PMMA that discussed above might also be related to its formation of longer trains.

## 4. Segmental Dynamics

In this section, we analyze the interface induced gradient of the segmental dynamics, and the overall dynamical properties of the confined melts. Furthermore, the segmental relaxation curves are shown in the frequency domain and some insights related to dielectric spectroscopy experiments are provided.

### 4.1. Mobility Gradient

The backbone torsional motions of PMMA chains are closely related to their segmental α -relaxation [46,47,48]. Therefore, we study segmental dynamics through the calculation of torsional autocorrelation function (TACF) for backbone dihedral angles of PMMA stereoisomers. TACF is defined as [47]:

(1) $$\mathrm{TACF}\left(t\right)=\frac{\langle \cos\phi \left(t\right)\cos\phi \left(0\right)\rangle -{\langle \cos\phi \left(0\right)\rangle }^{2}}{\langle \cos^{2}\phi \left(0\right)\rangle -{\langle \cos\phi \left(0\right)\rangle }^{2}}$$

where ϕ(t) and ϕ(0) are backbone dihedral angles at times *t* and 0, and 〈〉 shows averaging over all appropriate dihedral angles and time origins. TACF is calculated both for the layers at different distances from the nanosheets (layer resolved approach) and for the entire confined systems (averaged over all backbone dihedral angles in the system).

For the layer resolved calculation of TACF, confined films are divided into five layers: the closet layer to the surface ( [0 – 7] Å) corresponds to the adsorption layer that was observed in $f\left(d\right)/f^{\circ }$ curves (Figure 2b). At the second layer ( [7 – 15] Å), PMMA chains show slight perturbations of local density (particularly i-PMMA chains) and the other three layers ( [15 – 25] Å, [25 – 35] Å, and [35 – 50] Å) concern regions where the interfacial layering effects are almost negligible (Figure 2b). Each dihedral angle contributes to the TACF of a layer based on the its position (minimum distance from the middle point between the central atoms of the dihedral angle to the surface atoms) at the time origin. One can think of an alternative approach in which a dihedral angle contributes to the TACF of a layer for times less than its residence time in that layer (this scheme was used in our previous calculation of TACF for PMMA/PG systems [49]). Here, we have chosen the former scheme because, by following it: (i) the results are totally independent of the saving frequency of the coordinates, (ii) the residence times of segments in each layer is implicitly taken into account, and above all (iii) one can write the autocorrelation function of the entire system as a simple linear combination of the autocorrelation functions of the layers (see Section 4.3 and Equation (4)).

**Figure 3:**
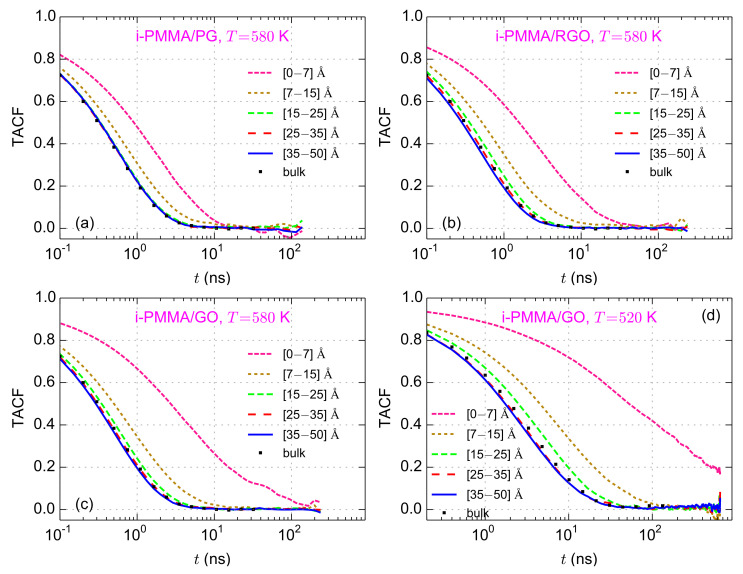
Layer resolved torsional autocorrelation function (TACF) curves for (**a**) i-PMMA/PG; (**b**) i-PMMA/RGO; (**c**) i-PMMA/GO at T=580 K; and (**d**) i-PMMA/GO at T=520 K.

The layer resolved TACF curves for i-PMMA melts confined between PG, RGO, and GO, at T=580 K, are plotted in Figure 3a–c; the TACF curves for i-PMMA/GO system at T=520 K are depicted in Figure 3d (the layer resolved TACF curves for the confined i-PMMA melts at T=550 K and some of the model s-PMMA containing confined systems are provided in Figures S1 and S2 of the Supplementary Materials). For all model systems, a *gradient of segmental mobility* is observed. TACF curves for PMMA segments being closer to the model surfaces decay slower than those that are far away. The decelerations of the backbone torsional motions for the first and the second studied layers, i.e., up to 1.5 nm from the surfaces, are significant. In addition, for the PMMA chains near GO and RGO, even at the third layer (distances between 1.5 nm to 2.5 nm) the segmental dynamics is slightly slower than that of the bulk melt. At the center of the films, the torsional mobility is rather close to that of the respective bulk melts. To quantify the mobility gradient at the interface, the TACF curve of each layer has been fitted with a stretched exponential, Kohlrausch–Williams–Watts (KWW), function and the relaxation (or correlation) time of the backbone torsional motions, $\tau_{\mathrm{seg}}$ , has been calculated as the integral of the KWW function. The KWW function is defined as [47]:

(2) $$f\left(t\right)=A\exp[-{\left(\frac{t}{\tau_{\mathrm{KWW}}}\right)}^{\beta }]$$

where A≤1 is a prefactor that takes into account the fast vibrational and librational motions, $\tau_{\mathrm{KWW}}$ is the characteristic time of the KWW function, and β≤1 is the stretch exponent.

**Figure 4:**
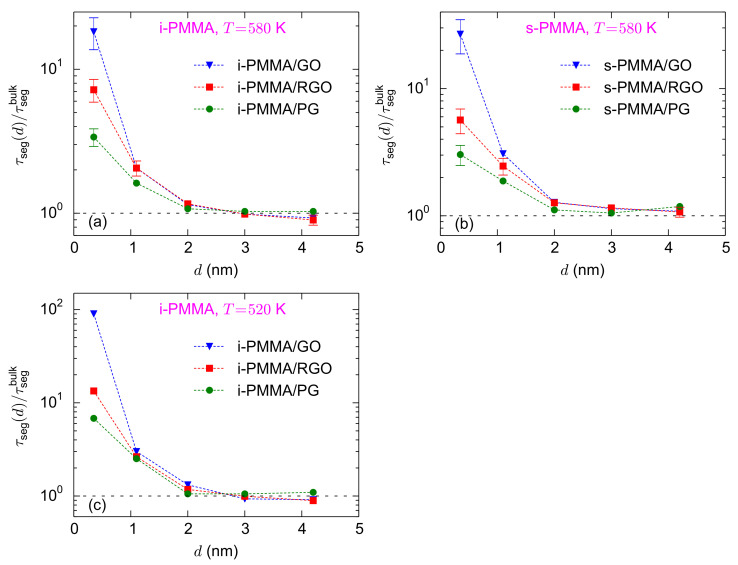
Interfacial segmental relaxation times, normalized with the respective bulk values, $\tau_{\mathrm{seg}}\left(d\right)/\tau_{\mathrm{seg}}^{\mathrm{bulk}}$ , at different distances from the nanosheets: (**a**–**c**) show the results for confined i-PMMA at T=580 K, confined s-PMMA at T=580 K, and confined i-PMMA at T=520 K, respectively. Each panel shows the surface–chemistry dependence of $\tau_{\mathrm{seg}}\left(d\right)/\tau_{\mathrm{seg}}^{\mathrm{bulk}}$ . The dashed lines are guides to the eye. The error bars at T=580 K were estimated based on the block averaging method.

Figure 4a–c shows the surface–chemistry dependence of the interfacial segmental relaxation times, normalized with the respective bulk values, for confined i-PMMA at T=580 K, confined s-PMMA at T=580 K, and confined i-PMMA at 520 K. In the vicinity of the three model surfaces, backbone torsional mobilities of PMMA chains slow down. However, the larger the concentration of functional groups, the slower the interfacial dynamics. For the adsorption (closest) layer, at T=580 K, the values of $\tau_{\mathrm{seg}}/\tau_{\mathrm{seg}}^{\mathrm{bulk}}$ are 3.4 , 7.2 , and 18.2 for i-PMMA/PG, i-PMMA/RGO, and i-PMMA/GO, respectively (Figure 4a). Qualitatively similar surface–chemistry dependence of the segmental dynamics was observed for all PMMA stereoisomers at all temperatures; in all cases, in the interfacial region: $\tau_{\mathrm{seg}}^{\mathrm{GO}}>\tau_{\mathrm{seg}}^{\mathrm{RGO}}>\tau_{\mathrm{seg}}^{\mathrm{PG}}$ . Mainly due to the favorable electrostatic interactions of carbonyl oxygen atoms of PMMA with the hydroxyl functional groups of RGO and GO, the interfacial torsional dynamics is slower close to the RGO and GO surfaces, as compared to the PG surface.

**Figure 5:**
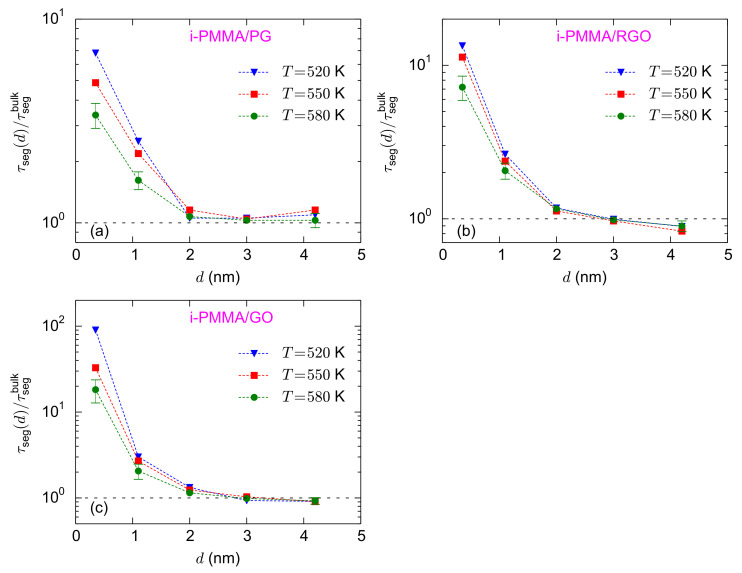
(**a**–**c**) The temperature dependence of $\tau_{\mathrm{seg}}\left(d\right)/\tau_{\mathrm{seg}}^{\mathrm{bulk}}$ for the i-PMMA/PG, i-PMMA/RGO, and i-PMMA/GO interfacial systems, respectively.

Figure 5a–c shows the temperature dependence of the normalized interfacial segmental relaxation times for the i-PMMA/PG, i-PMMA/RGO, and i-PMMA/GO systems. At the interfacial region (distances less than 2 nm), the values of $\tau_{\mathrm{seg}}/\tau_{\mathrm{seg}}^{\mathrm{bulk}}$ increase with decreasing temperature. For the adsorption layer of the i-PMMA/GO system, the values of $\tau_{\mathrm{seg}}/\tau_{\mathrm{seg}}^{\mathrm{bulk}}$ are 18.1 , 32.0 , and 90.0 at 580 K, 550 K, and 520 K, respectively (Figure 5c). The increase of $\tau_{\mathrm{seg}}\left(d\right)/\tau_{\mathrm{seg}}^{\mathrm{bulk}}$ , for small values of *d*, with decreasing temperature shows the stronger temperature dependence of the interfacial segmental dynamics as compared to dynamics of the respective bulk melts, for the temperature range studied here, well above the bulk *T_g_*. The temperature dependence of the interfacial dynamics for the s-PMMA/PG, s-PMMA/RGO, and s-PMMA/GO systems are provided in Figure S3 (of the Supplementary Materials); it seems that the temperature dependence of s-PMMA containing interfacial systems is qualitatively similar to that of i-PMMA containing ones; however, the results for the former cases are rather scattered and their temperature dependency is not as clear as the results of i-PMMA systems.

### 4.2. Mobility Gradient in Terms of a Temperature Shift

In the above analysis, we have described the surface induced gradient of the segmental dynamics in terms of $\tau_{\mathrm{seg}}\left(d\right)/\tau_{\mathrm{seg}}^{\mathrm{bulk}}$ . In the following, we express the gradient of the segmental dynamics in terms of a *temperature shift*, which is defined as below:

(3) $$\begin{array}{ccc} & \Delta T_{\mathrm{seg}}\left(d\right) & =T-T^{★}\left(d\right)\\ & T & :\mathrm{temperaure}\;\mathrm{of}\;\mathrm{the}\;\mathrm{system}\\ & T^{★}\left(d\right) & :\mathrm{such}\;\mathrm{that}\;\tau_{\mathrm{seg}}^{\mathrm{bulk}}\left(T^{★}\right)=\tau_{\mathrm{seg}}^{\mathrm{confined}}\left(T,d\right) \end{array}$$

$\Delta T_{\mathrm{seg}}\left(d\right)$ shows the shift (reduction) of temperature that should be considered for the bulk melt to make its (average) segmental relaxation time equal to the (average) segmental relaxation time of the confined melt at distance *d* from the confining surface. $\Delta T_{\mathrm{seg}}\left(d\right)$ , as compared to $\tau_{\mathrm{seg}}\left(d\right)/\tau_{\mathrm{seg}}^{\mathrm{bulk}}$ , offers an intuitive measure for the expression of the surface induced gradient of the segmental dynamics, and for the comparison between the interfacial segmental dynamics of two different polymers with different bulk *T_g_*s. Furthermore, as will be discussed below, $\Delta T_{\mathrm{seg}}\left(d\right)$ has a much weaker temperature dependence than $\tau_{\mathrm{seg}}\left(d\right)/\tau_{\mathrm{seg}}^{\mathrm{bulk}}$ . Note that, in some experimental works [3,10], the alterations of segmental dynamics have been expressed in terms of a shift in a characteristic temperature of the segmental dynamics (e.g., a shift in *T_g_*). $\Delta T_{\mathrm{seg}}$ is not precisely a shift of a characteristic temperature because it is not defined based on a specific relaxation time (for example, usually *T_g_* is measured as the temperature at which the relaxation time of the segmental process equals 100 s). However, at the studied temperature range, $\Delta T_{\mathrm{seg}}$ can be considered as an estimate of the shift of a characteristic temperature which is defined based on a relaxation time of around 1 ns to 1μ s (near the relaxation times of the model systems at the studied temperatures).

**Figure 6:**
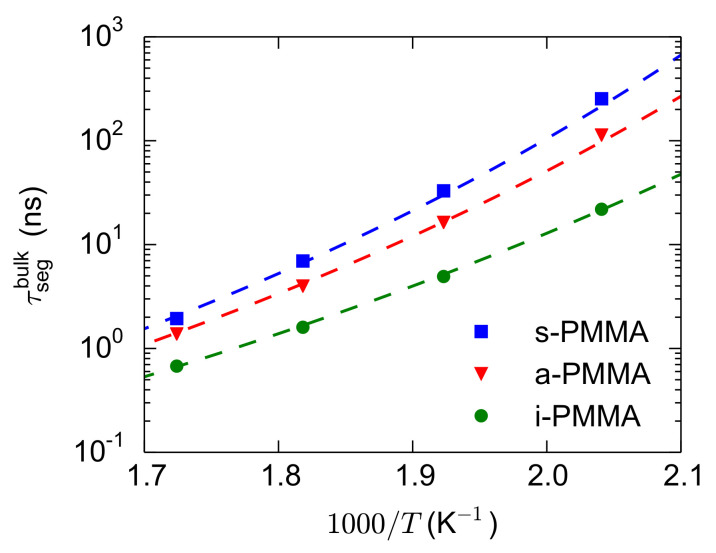
Relaxation times of the backbone torsional motions for the bulk melts of the model PMMA stereoisomers at different temperatures. The dashed lines are the fitted VFT curves on the data, used either for interpolation or for extrapolation over short temperature intervals.

For the calculation of $\Delta T_{\mathrm{seg}}$ , the bulk melts of i-, a-, and s-PMMA were simulated at 580 K, 550 K, 520 K, and 490 K and the $\tau_{\mathrm{seg}}^{\mathrm{bulk}}$ values were calculated. Then, the $\tau_{\mathrm{seg}}^{\mathrm{bulk}}$ values of each stereoisomer were fitted with a Vogel–Fulcher–Tammann (VFT) relation ( $\tau =\tau_{\infty }\exp\left(B/\left(T-T_{0}\right)\right)$ ). The parameters of the fitted VFT curves are: $\tau_{\infty }=10^{-13}$ sec and B=2800 K for the three model PMMA stereoisomers and $T_{0}=262$ K, 287 K, and 298 K for i-, a-, and s-PMMA, respectively (because of the extensions of the runs and recalculations of the relaxation times, the $T_{0}$ values are very slightly different from our previous results [44,49]). The bulk segmental relaxation times and the fitted VFT curves are shown in Figure 6. The obtained VFT relations are used for the estimation (through interpolation or extrapolation over short temperature intervals) of the temperature ( $T^{★}$ in Equation (3)) corresponding to a given relaxation time ( $\tau_{\mathrm{seg}}^{\mathrm{confined}}\left(T,d\right)$ in Equation (3)).

**Figure 7:**
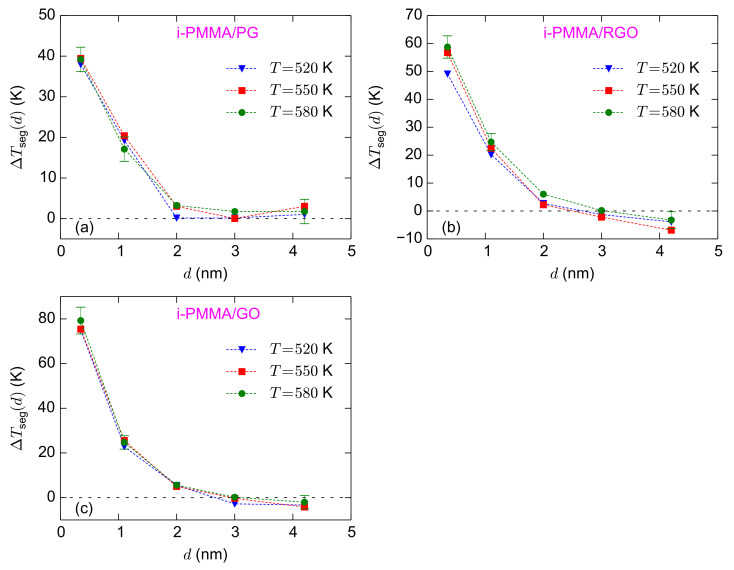
$\Delta T_{\mathrm{seg}}\left(d\right)$ for the confined i-PMMA melts at different temperatures; (**a**–**c**) show the results for i-PMMA/PG, i-PMMA/RGO, and i-PMMA/GO systems, respectively. The estimates of error bars at T=580 K are provided based on block averaging. At lower temperatures, larger error bars are expected.

Figure 7a–c shows the calculated values of $\Delta T_{\mathrm{seg}}\left(d\right)$ for the i-PMMA/PG, i-PMMA/RGO, and i-PMMA/GO systems, at all temperatures studied here. Note that Figure 7 is the representation of Figure 5 in terms of $\Delta T_{\mathrm{seg}}\left(d\right)$ . Figure 7 clearly shows that unlike $\tau_{\mathrm{seg}}\left(d\right)/\tau_{\mathrm{seg}}^{\mathrm{bulk}}$ , $\Delta T_{\mathrm{seg}}\left(d\right)$ has a weak temperature dependence, well above *T_g_*, and stays almost constant upon the variation of temperature. The observed trends for the temperature-dependences of $\tau_{\mathrm{seg}}\left(d\right)/\tau_{\mathrm{seg}}^{\mathrm{bulk}}$ and $\Delta T_{\mathrm{seg}}\left(d\right)$ deserve a comment. If we assume that the temperature dependence of the relaxation times at each interfacial layer (layers defined for the layer resolved analysis) can be described by a VFT relation, then the temperature-dependences can be rationalized: $\tau_{\mathrm{seg}}\left(d\right)/\tau_{\mathrm{seg}}^{\mathrm{bulk}}$ is the ratio of two exponential (VFT) functions and is expected to have an exponential temperature-dependence too. On the other hand $\Delta T_{\mathrm{seg}}\left(d\right)$ is expected to have a weaker temperature dependence: if the VFT relations of the bulk melt and the interfacial layers have similar values of $\tau_{\infty }$ (which seems reasonable), then $\Delta T_{\mathrm{seg}}$ of a layer becomes a linear function of temperature with a slope of $\left(1-B^{\mathrm{bulk}}/B^{\mathrm{layer}}\right)$ (*B* is the generalized activation energy of VFT function) (see Equation (3)); in the case of similar generalized activation energies $B^{\mathrm{bulk}}=B^{\mathrm{layer}}$ , $\Delta T_{\mathrm{seg}}$ becomes temperature independent.

Figure 7a–c also shows the surface–chemistry dependence of $\Delta T_{\mathrm{seg}}\left(d\right)$ (by comparing (a), (b), and (c) subplots). For the adsorption layer, $\Delta T_{\mathrm{seg}}$ approximately equals 39 K, 58 K, and 78 K for i-PMMA/PG, i-PMMA/RGO, and i-PMMA/GO, respectively; these differences reflect the slower dynamics of i-PMMA close to the oxidized surfaces. For the second studied layer ( 0.7 – 1.5 nm), the $\Delta T_{\mathrm{seg}}$ values fall in the interval of 17–25 K. The effect of surface–chemistry is less pronounced at the second layer relative to the first layer and $\Delta T_{\mathrm{seg}}$ of the second layer is almost similar for the RGO and GO containing systems (see also Table 1). The $\Delta T_{\mathrm{seg}}$ values at the center of the i-PMMA/RGO and i-PMMA/GO confined systems are also worth attention; it seems that they are systematically negative (the behavior is seen at all temperatures) revealing slightly faster segmental dynamics relative to the bulk melt (see also Figure 5 and Table 1). This behavior is also seen for a-PMMA/RGO and a-PMMA/GO systems (Table 1); however, s-PMMA or PG containing systems do not exhibit it.

**Figure 8:**
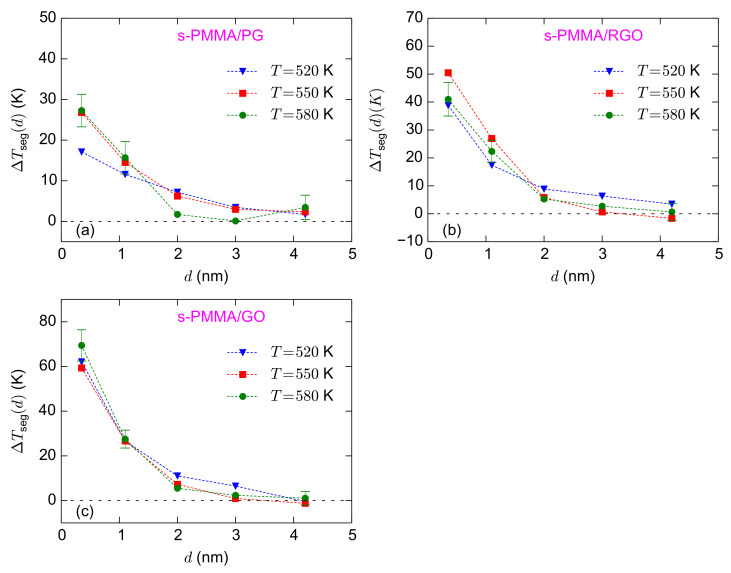
$\Delta T_{\mathrm{seg}}\left(d\right)$ for the confined s-PMMA melts at different temperatures; (**a**–**c**) show the results for s-PMMA/PG, s-PMMA/RGO, and s-PMMA/GO systems, respectively. The estimates of error bars at T=580 K are provided based on block averaging. At lower temperatures, larger error bars are expected.

The calculated values of $\Delta T_{\mathrm{seg}}\left(d\right)$ for the s-PMMA confined systems are provided in Figure 8. Comparing different temperatures, the $\Delta T_{\mathrm{seg}}\left(d\right)$ values of s-PMMA systems are more scattered than those of i-PMMA ones. However, the general trend seems similar to the results of i-PMMA systems and, in the studied temperature range, $\Delta T_{\mathrm{seg}}\left(d\right)$ seems to have a weak temperature dependence. For the adsorption layer, $\Delta T_{\mathrm{seg}}$ approximately equals 27 K, 41 K, and 69 K for s-PMMA/PG, s-PMMA/RGO, and s-PMMA/GO, respectively (based on T=580 K data). For all cases, at the adsorption layer, interfacial s-PMMA systems have lower values of $\Delta T_{\mathrm{seg}}$ relative to the corresponding i-PMMA systems. This trend of the interfacial segmental dynamics correlates with the better interfacial packing of, and the formation of longer trains as well, by i-PMMA chains, as compared to s-PMMA ones (see Figure 2).

To summarize the surface–chemistry and tacticity effects, we present in Table 1 the layer resolved values of $\Delta T_{\mathrm{seg}}$ for i-, a-, and s-PMMA melts confined between PG, RGO, GO nanosheets; the data of Table 1 are calculated based on the simulations at 580 K. With increasing the concentration of the surface functional groups, $\Delta T_{\mathrm{seg}}$ increases at distances less than 2.5 nm (the first three layers). Particularly, at the adsorption layer ( [0 – 0.7] nm): $\Delta T_{\mathrm{seg}}^{\mathrm{GO}}>\Delta T_{\mathrm{seg}}^{\mathrm{RGO}}>\Delta T_{\mathrm{seg}}^{\mathrm{PG}}$ ; at the second and third layers: $\Delta T_{\mathrm{seg}}^{\mathrm{GO}}\approx \Delta T_{\mathrm{seg}}^{\mathrm{RGO}}>\Delta T_{\mathrm{seg}}^{\mathrm{PG}}$ . The effect of PMMA stereochemistry is also mainly seen at the adsorption layer; close to the three model surfaces, i-PMMA has a higher $\Delta T_{\mathrm{seg}}$ than s-PMMA and a-PMMA. As mentioned above, this behavior is consistent with the formation of tacticity dependent interfacial structures. It seems that a-PMMA and s-PMMA have comparable values of $\Delta T_{\mathrm{seg}}$ at the interfacial region (the differences are within the error bars).

**Figure 9:**
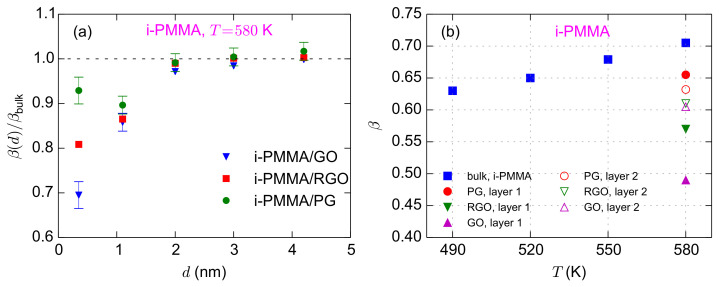
(**a**) Layer resolved values of KWW exponent ( β ) normalized with the bulk value for i-PMMA/PG, i-PMMA/RGO, and i-PMMA/GO systems at T=580 K. (**b**) Values of β for the first and second interfacial layers (0–7Å and 7–15Å) at T=580 K are shown together with the bulk values of β at different temperatures.

In the last part of this section, we discuss the values of the KWW exponents ( β in Equation (2)) for the confined systems. In general, a lower value of β corresponds to a broader distribution of relaxation times in the system. The layer resolved values of β for i-PMMA/PG, i-PMMA/RGO, and i-PMMA/GO systems at T=580 K are provided in Figure 9a. The interfacial layers, particularly the first and second layers, have lower values of β relative to the bulk melts; furthermore, increasing the concentration of functional groups leads to the decrease of β at the interfacial region. Above, we have described the deceleration of the segmental dynamics at the interfacial region through a temperature shift ( $\Delta T_{\mathrm{seg}}$ ). Thus, it is reasonable to compare the β of an interfacial layer (at distance *d*) with the β of the bulk melt at a lower temperature, where the interfacial layer and the bulk melt have a similar average segmental relaxation time. Figure 9b shows the values of β for the the first and second interfacial layers for i-PMMA confined systems at T=580 K, together with the values of β for the bulk melts of i-PMMA at different temperatures. From Table 1, one can find the temperature shifts that make the correlation times of the bulk melt similar to the correlation times of the interfacial layers. As shown in Figure 9b, with decreasing temperature, β of the bulk system decreases (this is to be expected since as a system approaches *T_g_* its dynamics becomes more heterogeneous); however, in all i-PMMA/PG, i-PMMA/RGO, and i-PMMA/GO cases, segments in both the first and second interfacial layers exhibit lower β values than the corresponding bulk segments with the same (average) segmental relaxation time. Therefore, it is clear that the distribution of the segmental relaxation times is broader in the interfacial layers, even when the average relaxation times of the bulk melt and the interfacial layers are similar.

### 4.3. Overall Dynamics of the Confined Melts

**Figure 10:**
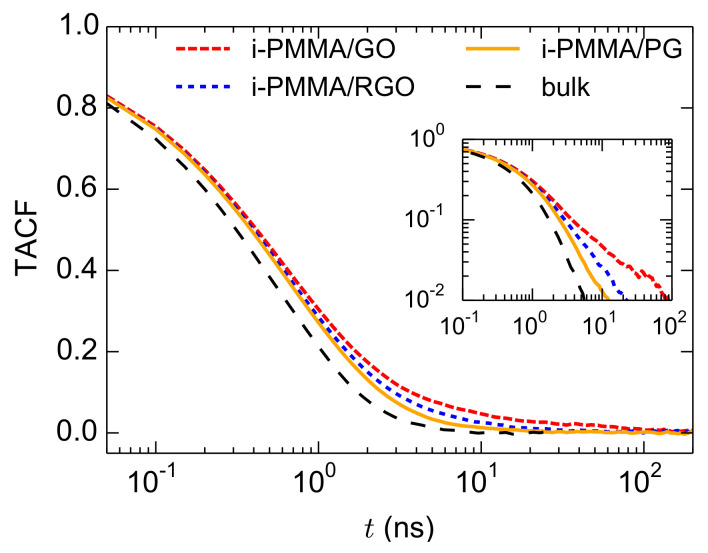
Overall TACF curves (calculated for the entire confined melts) for i-PMMA/PG, i-PMMA/RGO, and i-PMMA/GO systems at T=580 K. The inset shows the data in a log-log plot.

Having presented the layer resolved gradients of the segmental dynamics, here, we discuss the overall (average over the entire system) segmental dynamics of the confined PMMA melts. This is particularly important since in experiments a layer-resolved analysis is not straightforward, and usually the average segmental dynamics of nanocomposites and thin films is detected. The TACF curves, calculated over the entire confined melts, for i-PMMA chains confined between the PG, RGO, and GO nanosheets, at T=580 K, are provided in Figure 10; the corresponding bulk curve is also shown. As expected, the TACF curves of the confined melts decay slower than that of the respective bulk curve due to the contributions of the slower segments in the vicinity of the model nanosheets. In addition, with increasing the concentration of the surface functional groups, the decay of TACF becomes slower. The TACF curves of the confined melts have long time tails and they can not be fitted with a single KWW function; even the TACF of the PMMA/PG systems can not be perfectly fitted with a single KWW function. The issue is discussed below.

As mentioned before, for the layer resolved calculation of TACF, each dihedral angle counted in the TACF of a layer based on the its position at the time origin. Due to the use of this scheme for the calculation of layer resolved TACF, and because the denominator of Equation (1) is almost similar for different layers, the overall TACF of the confined melts can be separated into TACF of different layers:

(4) $${\mathrm{TACF}}^{\mathrm{overall}}\left(t\right)=\sum_{i=1}^{N}\phi_{i}\;{\mathrm{TACF}}_{i}\left(t\right)$$

where *N* is the number of layers, ${\mathrm{TACF}}_{i}\left(t\right)$ is the TACF (t) of the $i^{\mathrm{th}}$ layer, and $\phi_{i}$ is the fraction of backbone dihedral angles that belong to the $i^{\mathrm{th}}$ layer. This relation clearly shows the contributions of different layers in the overall TACF of the confined melts. The segmental relaxations of the first two interfacial layers (particularly of the first layer, or the adsorption layer) are well separated from the middle layers (see Table 1); this explains the inadequacy of a single KWW function for fitting the overall TACF curves.

Considering Equation (4), it is informative to briefly discuss the fraction of backbone dihedral angles belonging to the adsorption (first) layer, $\phi_{1}$ . For the systems studied here, $\phi_{1}$ falls in the interval of 0.11 to 0.15 ; however, it has surface–chemistry and tacticity dependence and increases with the concentration of surface functional groups and population of isotactic sequences of PMMA (it does not have significant temperature dependence in the studied temperature range). The values of $\phi_{1}$ for i-PMMA/PG, i-PMMA/RGO, i-PMMA/GO are 0.117 , 0.133 , and 0.151 , respectively. The values of $\phi_{1}$ for i-PMMA/RGO, a-PMMA/RGO, and s-PMMA/RGO are 0.133 , 0.128 , and 0.128 , respectively. It is worth mentioning that the values of $\phi_{1}$ are close to the fractions of train monomers (or monomers belonging to the adsorption layer). The dependence of $\phi_{1}$ on surface–chemistry is consistent with the stronger interactions of RGO and GO, compared to PG, with PMMA and also their higher roughness which might increase the effective surface area (or available volume around surfaces) for the adsorption of monomers. The dependence of $\phi_{1}$ on PMMA tacticity is consistent the formation of longer trains by i-PMMA compared to a-, and s-PMMA (see Figure 2c). To provide a rough estimate for $\phi_{1}$ , one can calculate 2×δ/D where *D* is the confined film thickness, δ=0.7Å is the length scale of adsorption of monomers, and the coefficient 2 takes into account two sides of the model surfaces; here, in all cases D≈10 nm, so 2×0.7/10=0.14 (as mentioned above, actual values vary between 0.11 and 0.15).

### 4.4. Relaxation Spectra in the Frequency Domain

**Figure 11:**
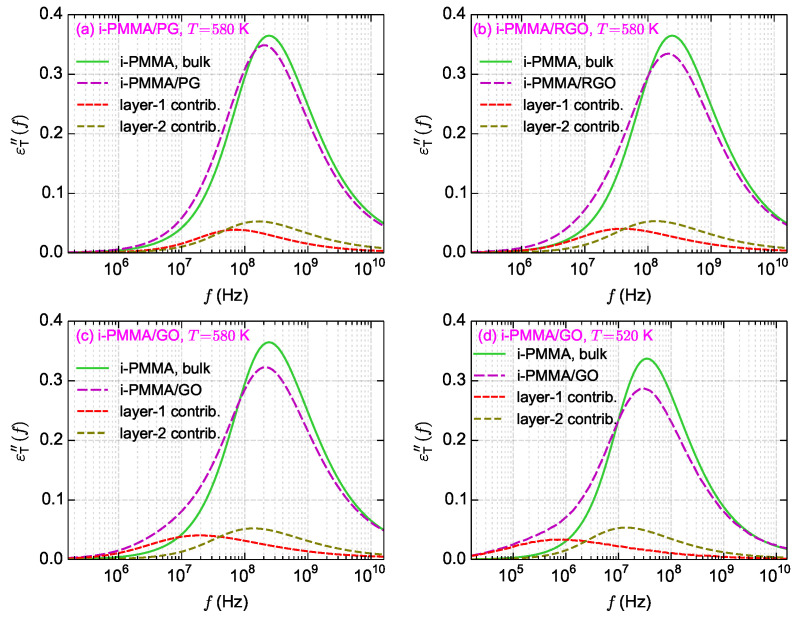
The loss parts of the backbone torsional autocorrelation functions. (**a**–**c**) The results for i-PMMA/PG, i-PMMA/RGO, and i-PMMA/GO systems at T=580 K, and (**d**) the results for i-PMMA/GO system at T=520 K. In all panels, the $\varepsilon_{T}^{{}^{\prime \prime }}\left(f\right)$ for the bulk and confined melts, and the contributions of the first and second interfacial layers in the $\varepsilon_{T}^{{}^{\prime \prime }}\left(f\right)$ of the confined melts, are shown. These curves in the time domain are presented in Figure 3 and Figure 10.

**Figure 12:**
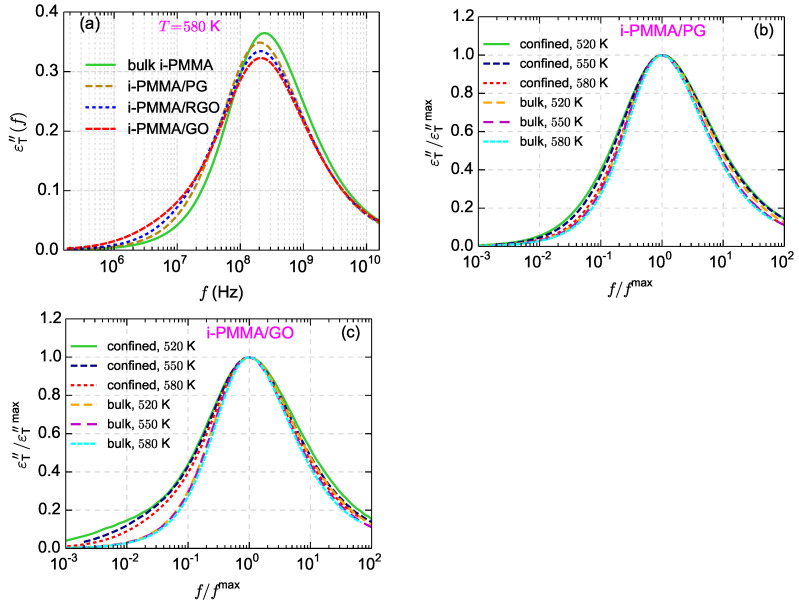
(**a**) Surface chemistry dependence of $\varepsilon_{T}^{{}^{\prime \prime }}\left(f\right)$ for the confined i-PMMA melts at T=580 K. (**b**,**c**) The $\varepsilon_{T}^{{}^{\prime \prime }}\left(f\right)$ spectra, normalized with the frequency and height of their maxima, for the confined i-PMMA/PG systems and confined i-PMMA/GO systems at different temperatures; the normalized spectra for the bulk i-PMMA melts are also presented.

It is informative to look at the TACF curves at the frequency domain to be able to qualitatively compare them to experimental results, for example the results of dielectric relaxation spectroscopy. Qualitative agreement between the TACF frequency spectra and α -relaxation spectra is expected since backbone torsional motions underlie the α -relaxation of PMMA. Analogous to the calculation of dielectric loss from dipole moment autocorrelation function [19,50,51], here we calculate the loss component of backbone torsional motions, $\varepsilon_{T}^{{}^{\prime \prime }}\left(f\right)$ , from the Fourier transform of TACF (t) ( $\varepsilon_{T}^{{}^{\prime \prime }}\left(f\right)=2\pi f\int_{0}^{\infty }\mathrm{TACF}\left(t\right)\cos\left(2\pi ft\right)dt$ , where *f* is frequency) [19]. Note that, for PMMA, both α -relaxation and β -relaxation processes (which are related to backbone and side-chain motions, respectively [46,47,48]) contribute to the decay of the dipole moment autocorrelation function; however, using the torsional autocorrelation function, we directly analyze the backbone motions. $\varepsilon_{T}^{{}^{\prime \prime }}\left(f\right)$ curves of the bulk melts and different layers (layers that were defined in the layer resolved analysis of TACF) were obtained by applying Fourier transform to the KWW fits of the corresponding TACF (t) curves. To calculate $\varepsilon_{T}^{{}^{\prime \prime }}\left(f\right)$ for the entire confined melts, we constructed a smooth curve for the TACF ${}^{\mathrm{overall}}\left(t\right)$ using KWW fits of all layers and Equation (4); then, we applied Fourier transform to that smooth curve. The Fourier transforms were calculated using a Filon-Trapezoidal rule [52] on equally spaced data in the time domain. Some of the calculated $\varepsilon_{T}^{{}^{\prime \prime }}\left(f\right)$ curves (loss components of TACF (t) for backbone dihedral angles) are shown in Figure 11 and Figure 12.

Figure 11a–c shows the calculated $\varepsilon_{T}^{{}^{\prime \prime }}\left(f\right)$ curves for i-PMMA/PG, i-PMMA/RGO, and i-PMMA/GO systems at T=580 K, whereas Figure 11d presents $\varepsilon_{T}^{{}^{\prime \prime }}\left(f\right)$ curves for i-PMMA/GO at T=520 K. In each panel of Figure 11, the $\varepsilon_{T}^{{}^{\prime \prime }}\left(f\right)$ curves of the bulk melt, the (entire) confined melt, and the contributions of the first and second interfacial layers (0–7 Å and 7–15 Å) in the $\varepsilon_{T}^{{}^{\prime \prime }}\left(f\right)$ of the confined melt are presented. In addition, Figure 12a explicitly shows the surface–chemistry dependence of $\varepsilon_{T}^{{}^{\prime \prime }}\left(f\right)$ for the confined melts of i-PMMA at T=580 K. Some important observations can be made out of these data. Due to the deceleration of the segmental dynamics at the interfacial region, the $\varepsilon_{T}^{{}^{\prime \prime }}\left(f\right)$ curves of the confined melts are broader (toward lower frequencies) and have lower amplitudes relative to the corresponding bulk curves. These effects become more pronounced with increasing the concentration of surface functional groups and decreasing temperature. The position of the peak maximum is also worth attention: in all cases, a slight shift of the maximum of the $\varepsilon_{T}^{{}^{\prime \prime }}\left(f\right)$ (of the confined melts) to lower frequencies is observed, whereas the shift is more pronounced in the case of PG containing systems. Figure 11c,d show the loss spectra of i-PMMA/GO system at T=580 K and T=520 K, respectively. With reducing temperature, the separation between the bulk and the interfacial segmental processes increases on the frequency axis (see also the data of Figure 5); hence, at 520 K, as compared to 580 K, a rather distinct shoulder is seen in the $\varepsilon_{T}^{{}^{\prime \prime }}\left(f\right)$ curve of the i-PMMA/GO system.

Figure 12b,c shows the temperature dependence of normalized $\varepsilon_{T}^{{}^{\prime \prime }}\left(f\right)$ spectra (normalized with the frequency and height of their maxima) for the bulk i-PMMA melts and the confined i-PMMA melts between PG and GO sheets; normalization has been done following the procedure used for checking the applicability of time-temperature superposition principle [51]. Upon normalization, the bulk spectra become almost identical (except slight differences in high frequency region). However, the shape of the normalized confined-melt spectrum is temperature dependent (particularly for i-PMMA/GO, Figure 12c) which shows the shortcoming of the time-temperature superposition for describing the segmental dynamics of the model confined melts [51]. The reason is the stronger temperature dependence of the segmental dynamics at the interfacial layer than the segmental dynamics at (rather bulk-like) middle layer (see also Figure 5).

It is worth comparing, qualitatively, the simulation results about the $\varepsilon_{T}^{{}^{\prime \prime }}\left(f\right)$ spectra to the experimental α -relaxation dielectric loss spectra of polymer nanostructured materials. The broadening of the dielectric loss spectrum toward low frequencies and reducing the strength of its peak have been observed for the α -relaxation spectra of nanocomposites containing attractive nanoparticles [8,9,10,11,14,53]. Furthermore, in some cases, a slight shift of the peak position has been observed [8,10,11,14]. In some experimental works, the loss spectra of nanocomposites were fitted with two Havriliak-Negami (HN) functions (corresponding to 2 KWW functions in the time domain), one function for the interfacial relaxation and another for the relaxation in the bulk-like region; [6,9,10] or they were fitted with an interfacial layer model assuming the presence of an interfacial phase in the system [10,11,14]. Using such procedures, the average relaxation time in the interfacial layer, the thickness of the interfacial layer, and the shift of *T_g_* at the interfacial layer have been estimated [6,9,10,11]. For example, for the interfacial layers of poly(styrene-*co*-butadiene)/silica, poly(2-vinyl pyridine)/silica, poly(vinyl acetate)/silica nanocomposites, 65 K [6], 8 K [9], and 6–8 K [10] increase of *T_g_* relative to the bulk value have been reported (*T_g_* has been measured as the temperature at which the relaxation time of the segmental motion equals 100 s [6,9]). Broader and lower dielectric loss spectra of nanocomposites, relative to the bulk melts, and inadequacy of a single HN function for fitting the spectra are consistent with our observations about the $\varepsilon_{T}^{{}^{\prime \prime }}\left(f\right)$ of the confined melts (see Figure 11). However, considering the simulation results about the mobility gradient close to the confining surfaces (see Figure 11 for the contributions of the first and second layers in the $\varepsilon_{T}^{{}^{\prime \prime }}\left(f\right)$ spectra and Table 1 for the calculated $\Delta T_{\mathrm{seg}}$ for different layers), the description of the segmental dynamics in terms of only two relaxation processes (bulk and interfacial processes) seems a rather simplified picture.

Usually, in dielectric experiments, the fraction of interfacial segments is calculated from the ratio of the dielectric amplitude of the segmental relaxation in the nanocomposite to the amplitude in the corresponding bulk melt [6,8,9,10]. Here, we perform an analogous analysis and calculate the *apparent* fraction of interfacial segments, $\phi_{\mathrm{int}}^{\mathrm{app}}$ , from the amplitudes of $\varepsilon_{T}^{{}^{\prime \prime }}\left(f\right)$ curves:

(5) $$\phi_{\mathrm{int}}^{\mathrm{app}}=1-\frac{\varepsilon_{T}^{{}^{\prime \prime }\max}\;\;\mathrm{confined}}{\varepsilon_{T}^{{}^{\prime \prime }\max}\;\;\mathrm{bulk}}$$

**Figure 13:**
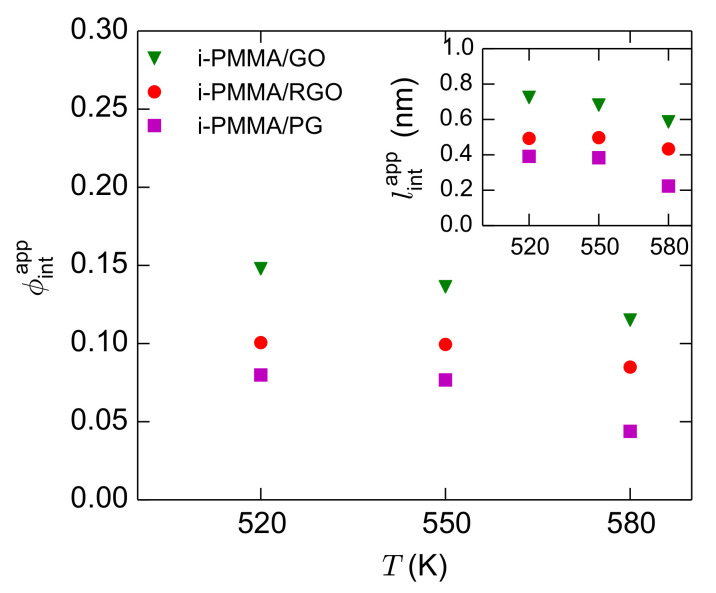
Apparent fraction of interfacial segments for the confined i-PMMA melts at different temperatures. The inset shows the apparent interface thickness.

In addition, from $\phi_{\mathrm{int}}^{\mathrm{app}}$ , the apparent interface thickness can be estimated through $l_{\mathrm{int}}^{\mathrm{app}}=\phi_{\mathrm{int}}^{\mathrm{app}}\times D/2$ , where *D* is the thickness of the confined film. The calculated values of $\phi_{\mathrm{int}}^{\mathrm{app}}$ and $l_{\mathrm{int}}^{\mathrm{app}}$ for the confined i-PMMA melts at different temperatures are shown in Figure 13. With decreasing temperature and increasing the concentration of the surface functional groups (increasing polymer/surface attraction), $\phi_{\mathrm{int}}^{\mathrm{app}}$ (and $l_{\mathrm{int}}^{\mathrm{app}}$ ) increases. The increase of $l_{\mathrm{int}}^{\mathrm{app}}$ with decreasing temperature has also been observed through dielectric spectroscopy experiments for polymer nanocomposites and thin films [10,14]. It is informative to look at the $\phi_{\mathrm{int}}^{\mathrm{app}}$ and $l_{\mathrm{int}}^{\mathrm{app}}$ data in the light of the microscopic layer resolved picture of the interfacial segmental dynamics that was provided before. Based on the layer resolved picture (Figure 3 and Figure 5), the spatial ranges of the gradients of the segmental dynamics close to the model surfaces stay almost constant upon decreasing temperature (at the temperature range studied here); furthermore, the spatial range of the gradient of the segmental dynamics is similar close to RGO and GO (the range is slightly shorter close to PG). Despite these underlying aspects, $\phi_{\mathrm{int}}^{\mathrm{app}}$ and $l_{\mathrm{int}}^{\mathrm{app}}$ increases with decreasing temperature and increasing the concentration of surface functional groups. Note that the actual fraction of interfacial segments (dihedral angles that to belong the interfacial layers) is also surface–chemistry dependent (see Section 4.3 for the values of the fraction of dihedral angles at the first layer, $\phi_{1}$ ); however, its surface–chemistry dependence is much weaker than that of $\phi_{\mathrm{int}}^{\mathrm{app}}$ . The way that $\phi_{\mathrm{int}}^{\mathrm{app}}$ is calculated explains these apparent discrepancies: $\phi_{\mathrm{int}}^{\mathrm{app}}$ shows the fraction of segments that exhibit a $\varepsilon_{T}^{{}^{\prime \prime }}\left(f\right)$ spectrum that is effectively separated from the bulk one ( $\varepsilon_{T}^{{}^{\prime \prime }}\;\mathrm{bulk}\left(f\right)$ ) on the frequency axis. Figure 11 clearly shows the meaning of $\phi_{\mathrm{int}}^{\mathrm{app}}$ ; for example, consider Figure 11a: near PG, the segmental dynamics at the first and second layers is slower than the bulk melt; the fractions of segments at theses layers are $\phi_{1}=0.117$ and $\phi_{2}=0.156$ . However, because at T=580 K, the $\varepsilon_{T}^{{}^{\prime \prime }}\left(f\right)$ spectra of theses layers are not well separated from the bulk spectrum on the frequency axis, $\phi_{\mathrm{int}}^{\mathrm{app}}=0.044$ , which is significantly lower than $\phi_{1}+\phi_{2}$ (even $\phi_{1}$ alone). Similarly, consider Figure 11c,d: close to GO, at both T=580 K and T=520 K, the first and second layers are significantly slower than the bulk melt (the third layer is also slightly slower than bulk). The fractions of segments located at these layers do not significantly changes with temperature ( $\phi_{1}\approx 0.15$ and $\phi_{2}\approx 0.17$ ). However, because reducing temperature leads to the separation of the interfacial and bulk $\varepsilon_{T}^{{}^{\prime \prime }}\left(f\right)$ spectra on the frequency axis, the increase of $\phi_{\mathrm{int}}^{\mathrm{app}}$ with decreasing temperature is observed; at 580 K and 520 K, $\phi_{\mathrm{int}}^{\mathrm{app}}=0.115$ and $\phi_{\mathrm{int}}^{\mathrm{app}}=0.148$ , respectively (at 520 K, $\phi_{\mathrm{int}}^{\mathrm{app}}\approx \phi_{1}$ , however still $\phi_{\mathrm{int}}^{\mathrm{app}}<\phi_{1}+\phi_{2}$ ). In short, at the studied temperature range, $\phi_{\mathrm{int}}^{\mathrm{app}}$ increases with decreasing temperature, not because the actual fraction of the interfacial segments increases, but because the loss spectrum of the interfacial segments becomes more separated from the bulk spectrum. These discussions about $\phi_{\mathrm{int}}^{\mathrm{app}}$ are useful for the interpretation of experimental dielectric spectroscopy results for thin films and nanocomposites.

## 5. Conclusions

Segmental dynamics in unentangled i-, a-, and s-PMMA melts confined between PG, RGO, or GO nanosheets was studied at different temperatures (520–580 K), well above *T_g_*, through atomistic molecular dynamics simulations. Segmental dynamics were studied through the calculation of torsional autocorrelation function for the backbone dihedral angles of PMMA chains. The backbone torsional motions were analyzed because they underlie the segmental α -relaxation of PMMA. The thicknesses of the model confined melts are around 10 nm and the PMMA chains are under rather moderate degree of confinement; i.e., $d/2R_{g}\approx 5$ , where *d* is the confined film thickness. The model RGO and GO nanosheets contain epoxide and hydroxyl functional groups, with different degrees of oxidations. The carbon to oxygen ratio is 3 for GO and 10 for RGO. Increasing the concentration of functional groups increases the attractive interaction of the nanosheets with PMMA, mainly due to the strong electrostatic interactions (hydrogen bonds) of the carbonyl functional groups of PMMA with the hydroxyl functional groups of the nanosheets. PMMA chains form tacticity-dependent interfacial structures; particularly, i-PMMA exhibits a better interfacial packing and forms longer trains than a-, and s-PMMA chains. The higher stiffness of i-PMMA chains than a-PMMA and s-PMMA ones can also explain the tacticity dependence of the size of trains. The main findings are summarized below:

(a) Gradients of the segmental dynamics were observed close to all model graphene-based nanosheets. In the vicinity of (particularly, at distances less than ≈2 nm form) the model confining surfaces, the backbone torsional mobility slows down. The effect becomes stronger with increasing the concentration of the functional groups (and hence increasing polymer/surface attraction and surface roughness). The spatial range of the gradient is slightly longer close to the RGO and GO surfaces relative to the PG one.

(b) Upon decreasing temperature, the ratios of the interfacial segmental relaxation times to the respective bulk relaxation times ( $\tau_{\mathrm{seg}}^{\mathrm{interface}}/\tau_{\mathrm{seg}}^{\mathrm{bulk}}$ ) increase, showing the stronger temperature dependence of the interfacial segmental dynamics relative to the bulk one.

(c) The surface-induced gradient of the segmental dynamics was described in terms of a temperature shift, $\Delta T_{\mathrm{seg}}\left(d\right)$ ; this quantity shows the shift (reduction) of temperature that should be applied to the bulk melt to make its (average) segmental relaxation time equal to the (average) segmental relaxation time of the confined melt at a distance *d* from the confining surface. Unlike $\tau_{\mathrm{seg}}^{\mathrm{interface}}/\tau_{\mathrm{seg}}^{\mathrm{bulk}}$ , $\Delta T_{\mathrm{seg}}\left(d\right)$ has a weak temperature dependence at the temperature range of this study (520–580 K, T> *T_g_*+ 100 K). For the adsorption layer, i.e., 0–0.7 nm from the confining surfaces, values of $\Delta T_{\mathrm{seg}}$ are around 79 K, 59 K, and 39 K, for i-PMMA/GO, i-PMMA/RGO, i-PMMA/PG, and 69 K, 41 K, and 27 K, for s-PMMA/GO, s-PMMA/RGO, and s-PMMA/PG systems, respectively. In all cases, at the adoption layer, confined s-PMMA melts have lower values of $\Delta T_{\mathrm{seg}}$ than the respective i-PMMA melts. This trend is in agreement with the better interfacial packing and longer trains of i-PMMA chains. For the second layer (0.7–1.5 nm), values of $\Delta T_{\mathrm{seg}}$ fall in the interval of 15–30 K.

(d) Interfacial layers have lower values of β (KWW exponent) relative to the corresponding bulk melts. Therefore, the distribution of relaxation times is wider in the interfacial layers. The distribution of the segmental relaxation times is broader in the interfacial layers, even when the bulk melt has a lower temperature such that the average relaxation times of the bulk melt and the interfacial layers are similar. The reduction of β at the interface becomes stronger with increasing the concentration of the surface functional groups.

(e) Analogous to the calculation of dielectric loss spectrum, we calculated the loss component of the torsional autocorrelation function for the backbone dihedral angles ( $\varepsilon_{T}^{{}^{\prime \prime }}\left(f\right)$ , where *f* is frequency) for the bulk melts, interfacial layers (layer-resolved approach), and entire confined melts. Because of the deceleration of the segmental dynamics at the interfacial region, the $\varepsilon_{T}^{{}^{\prime \prime }}\left(f\right)$ curves of the confined melts are broader (toward lower frequencies) and have lower amplitudes relative to the corresponding bulk curves; these effects become stronger with increasing the concentration of surface functional groups and decreasing temperature.

(f) The shapes of the normalized $\varepsilon_{T}^{{}^{\prime \prime }}\left(f\right)$ confined-melt spectra (normalized with respect to the position and height of their peaks) are not identical at different temperatures. The reason is the stronger temperature dependence of the segmental dynamics at the interfacial region compared to that of the segmental dynamics at the (rather bulk-like) middle region of the confined films.

(g) An apparent fraction of interfacial segments, $\phi_{\mathrm{int}}^{\mathrm{app}}$ , and an apparent interface thickness, $l_{\mathrm{int}}^{\mathrm{app}}$ , were calculated for each confined system from the ratio of the amplitudes of the confined and bulk $\varepsilon_{T}^{{}^{\prime \prime }}\left(f\right)$ curves. $\phi_{\mathrm{int}}^{\mathrm{app}}$ shows the fraction of segments for which their $\varepsilon_{T}^{{}^{\prime \prime }}\left(f\right)$ spectrum is effectively separated from the corresponding bulk spectrum on the frequency axis and increases with increasing the concentration of functional groups and decreasing temperature. $\phi_{\mathrm{int}}^{\mathrm{app}}$ increases with reducing temperature, not because the actual fraction of the interfacial segments increases, but because the loss spectrum of the interfacial segments becomes more separated from that of the bulk melt.

## Figures and Tables

**Table 1:** Layer resolved values of $\Delta T_{\mathrm{seg}}$ for i-, a-, and s-PMMA melts confined between PG, RGO, and GO nanosheets; the reported data are based on the simulations at T=580 K.

	$\Delta T_{\mathrm{seg}}\left(d\right)$ (K)				
	d∈[0 **–** 0.7] **nm**	[0.7 **–** 1.5] **nm**	[1.5 **–** 2.5] **nm**	[2.5 **–** 3.5] **nm**	[3.5 **–** 5] **nm**
i-PMMA/PG	39	17	3	2	2
i-PMMA/RGO	59±4	25	6	0	−3±3
i-PMMA/GO	79	25	5	0	−2
a-PMMA/PG	26	15	1	2	4
a-PMMA/RGO	53±6	24	6	−2	−5±3
a-PMMA/GO	65	25	7	0	−4
s-PMMA/PG	27	16	2	0	3
s-PMMA/RGO	41±6	22	5	3	1±3
s-PMMA/GO	69	27	6	2	1

## Data Availability

The data presented in this study are available within the article and its supplementary material.

## Supplementary Materials

The following are available at https://www.mdpi.com/2073-4360/13/5/830/s1.
