# Correction: Foroozani Behbahani, A.; Harmandaris, V. Gradient of Segmental Dynamics in Stereoregular Poly(methyl methacrylate) Melts Confined between Pristine or Oxidized Graphene Sheets. *Polymers* 2021, *13*, 830

**DOI:** 10.3390/polym14010106

**Published:** 2021-12-29

**Authors:** Alireza Foroozani Behbahani, Vagelis Harmandaris

**Affiliations:** 1 Institute of Applied and Computational Mathematics, Foundation for Research and Technology-Hellas, GR-71110 Heraklion, Greece; 2 Department of Mathematics and Applied Mathematics, University of Crete, GR-70013 Heraklion, Greece; 3 Computation-Based Science and Technology Research Center, The Cyprus Institute, 2121 Nicosia, Cyprus

The authors wish to make the following corrections to this paper: [1]. In Section 4.4 of the original publication, the torsional autocorrelation functions of the confined melts are shown on the frequency domain. The employed relation is provided in page 16 of the original publication. A factor of 2π has been omitted from that relation, and it should be replaced with: $\varepsilon_{T}^{{}^{\prime \prime }}\left(f\right)=2\pi f\int_{0}^{\infty }\mathrm{TACF}\left(t\right)\cos\left(2\pi ft\right)dt$, where *f* is frequency. In fact, in the original publication, the spectra are plotted as functions of angular frequency.

Because of the above mentioned 2π factor, the spectra shown in Figure 11, in Figure 12a, and in a part of the graphical abstract (Figure 11d is also presented there) should be horizontally shifted on the logarithmic scale. The updated figures are provided below. The rest of the paper’s figures and all discussions and conclusions remain unaltered. The original article has been updated.

## Figures and Tables

**Figure 11:**
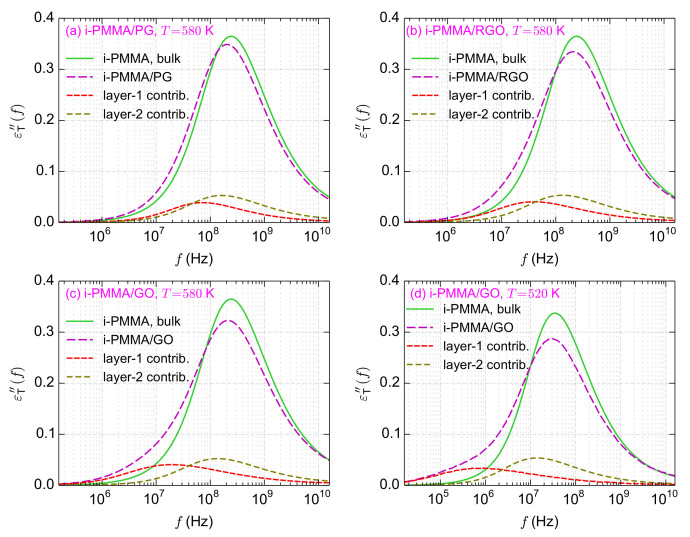
The loss parts of the backbone torsional autocorrelation functions. (**a**–**c**) The results for i-PMMA/PG, i-PMMA/RGO, and i-PMMA/GO systems at T=580 K, and (**d**) the results for i-PMMA/GO system at T=520 K. In all panels, the $\varepsilon_{T}^{{}^{\prime \prime }}\left(f\right)$ for the bulk and confined melts, and the contributions of the first and second interfacial layers in the $\varepsilon_{T}^{{}^{\prime \prime }}\left(f\right)$ of the confined melts, are shown. These curves in the time domain are presented in Figures 3 and 10.

**Figure 12a:**
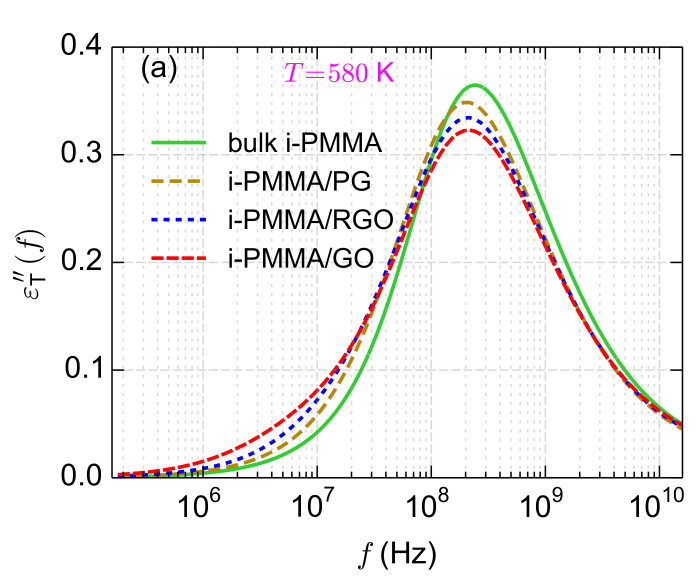
Surface chemistry dependence of $\varepsilon_{T}^{{}^{\prime \prime }}\left(f\right)$ for the confined i-PMMA melts at T=580 K.
