# Supplementary material for: Gradient of Segmental Dynamics in Stereoregular Poly(methyl methacrylate) Melts Confined between Pristine or Oxidized Graphene Sheets
Source: Polymers (Basel). 2021 Mar 8;13(5):830. doi: 10.3390/polym13050830 (PMC7962820; doi:10.3390/polym13050830)
Supplement: Supplementary file 1 [file polymers-13-00830-s001.pdf]

# Supplementary Materials for:

# Gradient of Segmental Dynamics in Stereoregular Poly(methyl methacrylate) Melts Confined Between Pristine or Oxidized Graphene Sheets

Alireza Foroozani Behbahani[1,*] and Vagelis Harmandaris[1,2,3,*]

[1] Institute of Applied and Computational Mathematics, Foundation for Research and Technology-Hellas, GR-71110 Heraklion, Greece

[2] Department of Mathematics and Applied Mathematics, University of Crete, GR-70013 Heraklion, Greece

[3] Computation-based Science and Technology Research Center, The Cyprus Institute, Nicosia 2121, Cyprus

Correspondence: af.behbahani@iacm.forth.gr; harman@uoc.gr

## S1 Force field

The force field parameters for the model graphene-based sheets and for PMMA are provided in Table S1 and Table S2. Both PMMA and graphene force fields exclude non-bonded interactions between the first and second chemically bonded neighbors. The Lorentz–Berthelot combination rule was used for calculating the Van der Waals interactions between dissimilar atoms (including interactions between PMMA and the model graphene-based nanosheets). More details about the force fields and their references are provided in the main text.

Table S1: Force field parameters for pristine and oxidized graphene sheets.

| non-bonded, $U_{\mathrm{LJ}}(r) = 4\varepsilon[(\frac{\sigma}{r})^{12} - (\frac{\sigma}{r})^6]$ + Coulomb | mass (g mol$^{-1}$) | $\sigma$ (nm) | $\varepsilon$ (kJ mol$^{-1}$) | $q$(e) |
|---|---|---|---|---|
| CGR | 12.011 | 0.3470 | 0.2750 | 0 |
| CEP | 12.011 | 0.3473 | 0.3979 | 0.2 |
| COH | 12.011 | 0.3473 | 0.3979 | 0.265 |
| OE | 15.9994 | 0.3033 | 0.4004088 | −0.4 |
| OH | 15.9994 | 0.3033 | 0.4004088 | −0.683 |
| H | 1.008 | 0.2846 | 0.063597 | 0.418 |
| bond stretching, Morse $U_{\mathrm{bond}} = D[1 - \exp(\beta(r - b_0))]^2$ | $b_0$ (nm) | $D$ (kJ mol$^{-1}$) | $\beta$ (nm$^{-1}$) | |
| CGR-CGR | 0.1418 | 478.9 | 21.867 | |
| bond stretching, Harmonic $U_{\mathrm{bond}} = \frac{1}{2}k_{\mathrm{bond}}(r - b_0)^2$ | $b_0$ (nm) | | $k_{\mathrm{bond}}$ (kJ mol$^{-1}$ nm$^{-2}$) | |
| CEP-CEP | 0.153 | | 292880.0 | |
| CEP-CGR | 0.146 | | 292880.0 | |
| CEP-COH | 0.153 | | 292880.0 | |
| CEP-OE | 0.142 | | 292880.0 | |
| CGR-COH | 0.146 | | 292880.0 | |
| COH-COH | 0.153 | | 292880.0 | |
| COH-OH | 0.142 | | 292880.0 | |
| H-OH | 0.098 | | 292880.0 | |
| angle bending, $U_{\mathrm{angle}} = \frac{1}{2}k_{\mathrm{angle}}(\cos\theta - \cos\theta_0)^2$ | $\theta_0$ (deg) | | $k_{\mathrm{angle}}$ (kJ mol$^{-1}$) | |
| CGR-CGR-CGR | 120 | | 562.2 | |
| *-CGR-*$^a$ | 120 | | 557.9 | |
| *-CEP-* | 109.471 | | 470.7 | |
| *-COH-* | 109.471 | | 470.7 | |
| CEP-OE-CEP | 104.51 | | 446.4 | |
| COH-OH-H | 104.51 | | 446.4 | |
| dihedral angle potential, $U(\phi) = k(1 + \cos(n\phi - \phi_0))$ | | | | |
| | $\phi_0$ (deg) | $k$ (kJ mol$^{-1}$) | n | |
| *-CGR-CGR-* | 180 | 12.56 | 2 | |
| *-CEP-CEP-* | 0 | 0.465 | 3 | |
| *-CEP-CGR-* | 180 | 0.349 | 6 | |
| *-CEP-COH-* | 0 | 0.465 | 3 | |
| *-CEP-OE-* | 0 | 1.39 | 3 | |
| *-CGR-COH-* | 180 | 0.349 | 6 | |
| *-COH-COH-* | 0 | 0.465 | 3 | |
| *-COH-OH-* | 0 | 1.395 | 3 | |

CGR: graphene $sp^2$ carbon atom. CEP: epoxide-group carbon atom. COH: hydroxyl-group carbon atom. OE: epoxide-group oxygen atom. OH: hydroxyl-group oxygen atom. H: hydroxyl-group hydrogen atom.
$a$: except CGR-CGR-CGR

Table S2: Force field parameters for PMMA.

| non-bonded, $U_{\mathrm{LJ}}(r) = 4\varepsilon[(\frac{\sigma}{r})^{12} - (\frac{\sigma}{r})^{6}]$ + Coulomb | mass (g mol$^{-1}$) | $\sigma$ (nm) | $\varepsilon$ (kJ mol$^{-1}$) | $q$(e) |
|---|---|---|---|---|
| CX (-$CH_3$ and -$CH_2$-) | 12.011 | 0.339967 | 0.457730 | 0.0051 |
| CX ($\alpha$ carbon) | 12.011 | 0.339967 | 0.457730 | 0.0189 |
| CX (ester-methyl) | 12.011 | 0.339967 | 0.457730 | 0.2801 |
| HC | 1.008 | 0.2649533 | 0.065689 | 0 |
| C | 12.011 | 0.339967 | 0.359824 | 0.7464 |
| O | 15.9994 | 0.2959922 | 0.878640 | $-0.5939$ |
| OS | 15.999 | 0.3000012 | 0.711280 | $-0.4617$ |
| H1 | 1.008 | 0.2471353 | 0.065689 | 0 |
| bond stretching, $U_{\mathrm{bond}} = \frac{1}{2}k_{\mathrm{bond}}(r - r_0)^2$ | $r_0$ (nm) | | $k_{\mathrm{bond}}$ (kJ mol$^{-1}$ nm$^{-2}$) | |
| CX-CX | 0.152 | | 259408.0 | |
| HC-CX | 0.1095 | | 301248.0 | |
| CX-C | 0.15 | | 276144.0 | |
| C-O | 0.1204 | | 835963.2 | |
| C-OS | 0.1343 | | 344175.9 | |
| OS-CX | 0.141 | | 267776.0 | |
| CX-H1 | 0.1092 | | 343088.0 | |
| angle bending, $U_{\mathrm{angle}} = \frac{1}{2}k_{\mathrm{angle}}(\theta - \theta_0)^2$ | $\theta_0$ (deg) | | $k_{\mathrm{angle}}$ (kJ mol$^{-1}$ rad$^{-2}$) | |
| HC-CX-HC | 109.5 | | 334.7 | |
| HC-CX-CX | 112.6 | | 376.6 | |
| CX-CX-CX | 113.5 | | 376.6 | |
| CX-CX-C | 111.5 | | 334.7 | |
| CX-C-O | 125.4 | | 493.7 | |
| CX-C-OS | 111.0 | | 418.4 | |
| C-OS-CX | 114.0 | | 423.4 | |
| O-C-OS | 122.5 | | 861.9 | |
| OS-CX-H1 | 110.0 | | 502.1 | |
| H1-CX-H1 | 109.5 | | 376.6 | |

| dihedral angle potential, $U(\phi) = \sum_{n=1}^{3} k_n(1 + \cos n\phi)$ | $k_1$ (kJ mol$^{-1}$) | $k_2$ | $k_3$ |
|---|---|---|---|
| CX-CX-CX-CX | 1.8828 | 0 | 0 |
| CX-CX-CX-HC | 0 | 0 | 0.4184 |
| CX-CX-CX-C | 0 | 0 | 0 |
| HC-CX-CX-C | 0 | 0 | $-0.12552$ |
| CX-CX-C-O | 0 | $-1.7154$ | $-1.2134$ |
| CX-CX-C-OS | 0 | $-1.8828$ | $-1.3807$ |
| O-C-OS-CX | 0 | $-17.61464$ | $-3.05432$ |
| H1-CX-OS-C | 0 | 0 | 0.6276 |
| CX-C-OS-CX | $-2.7196$ | $-11.506$ | 0 |

CX: $sp^3$ carbon atom. HC: hydrogen (except ester group hydrogen). C: $sp^2$ carbon atom.
O: carbonyl-group oxygen. OS: ester-group oxygen. H1: ester-side-group hydrogen.

## S2 Layer resolved TACF curves

The layer resolved torsional autocorrelation function (TACF) curves of the confined i-PMMA systems at $T = 550$ K are provided in Figure S1 and the curves for some of the model confined s-PMMA systems are provided in Figure S1.

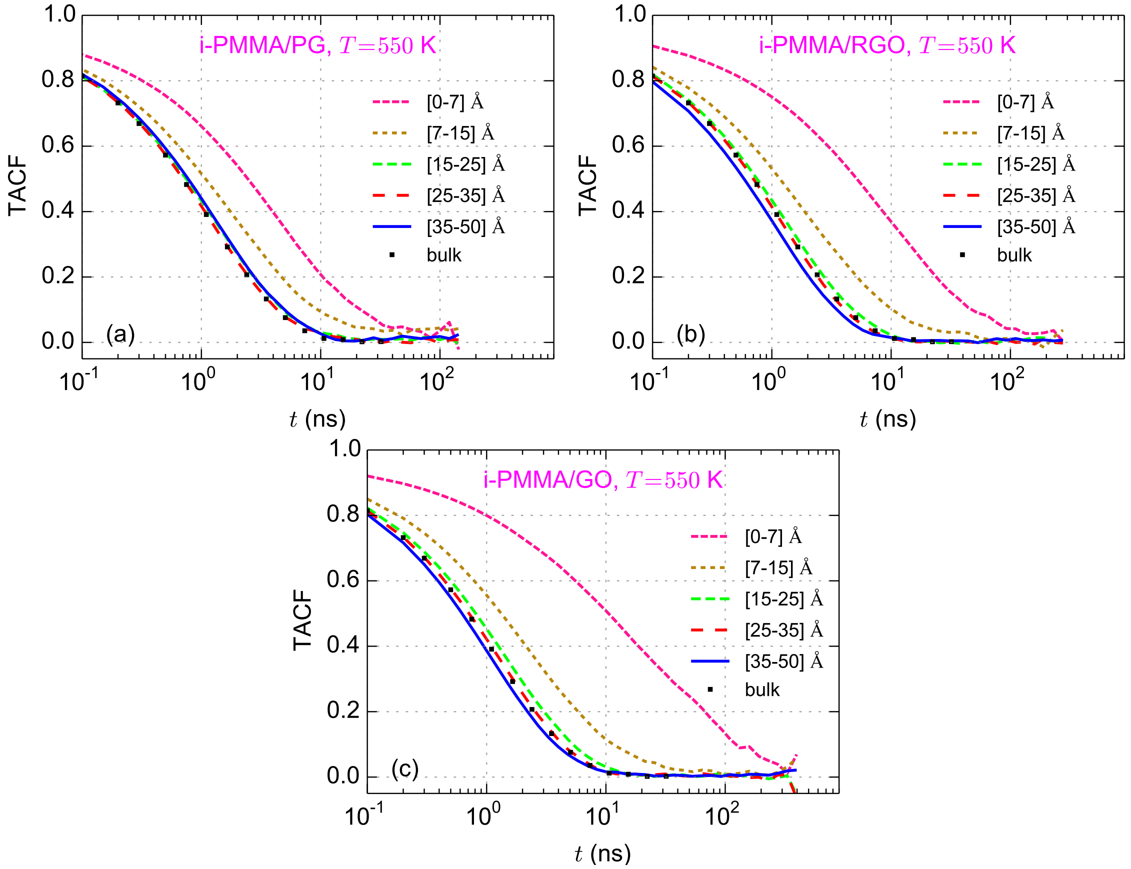


Figure S1: Layer resolved torsional autocorrelation function (TACF) curves for (a) i-PMMA/PG, (b) i-PMMA/RGO, and (c) i-PMMA/GO at $T = 550$ K.

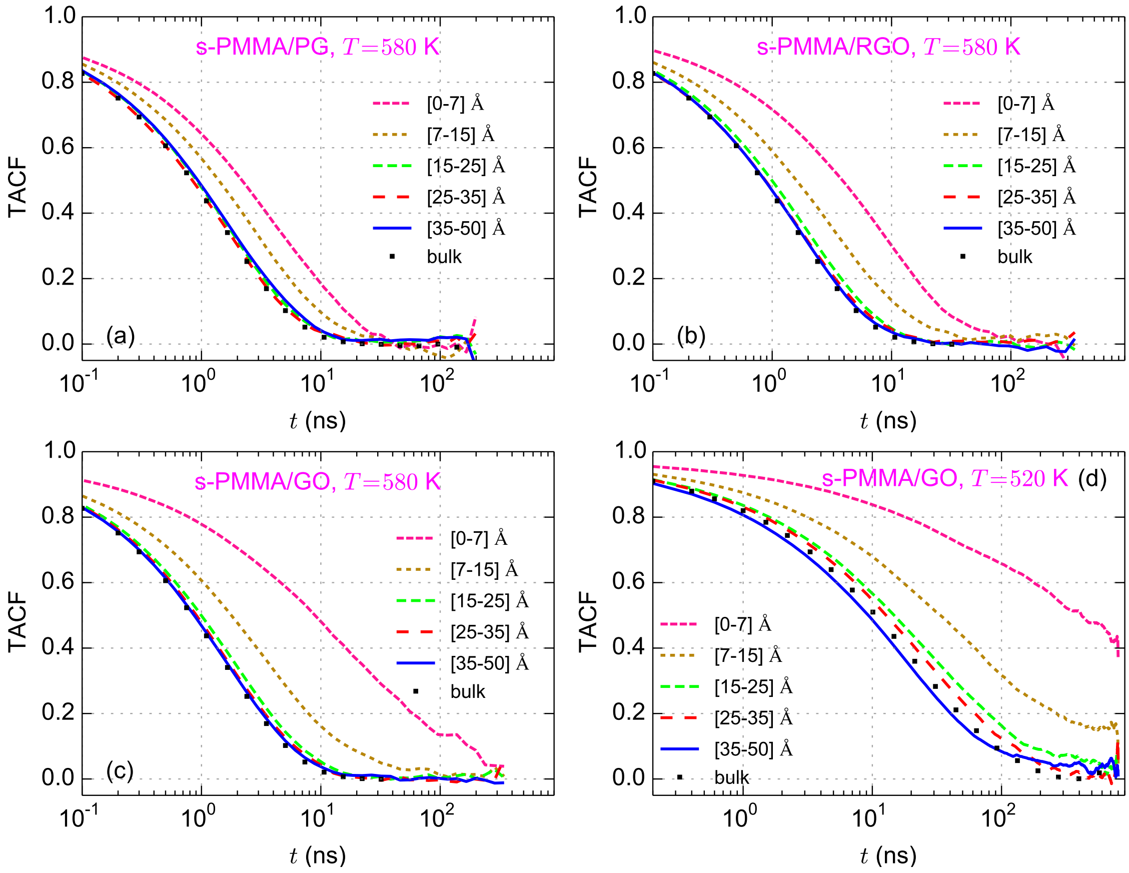


Figure S2: Layer resolved torsional autocorrelation function (TACF) curves for (a) s-PMMA/PG, (b) s-PMMA/RGO, (c) s-PMMA/GO at $T$ = 580 K, and (d) s-PMMA/GO at $T$ = 520 K.

## S3 Relaxation times of the confined s-PMMA systems

The layer resolved relaxation times of the s-PMMA/PG, s-PMMA/RGO. and s-PMMA/GO systems at different temperatures are provided in Figure S3. The relaxation times are normalized with the respective bulk values.

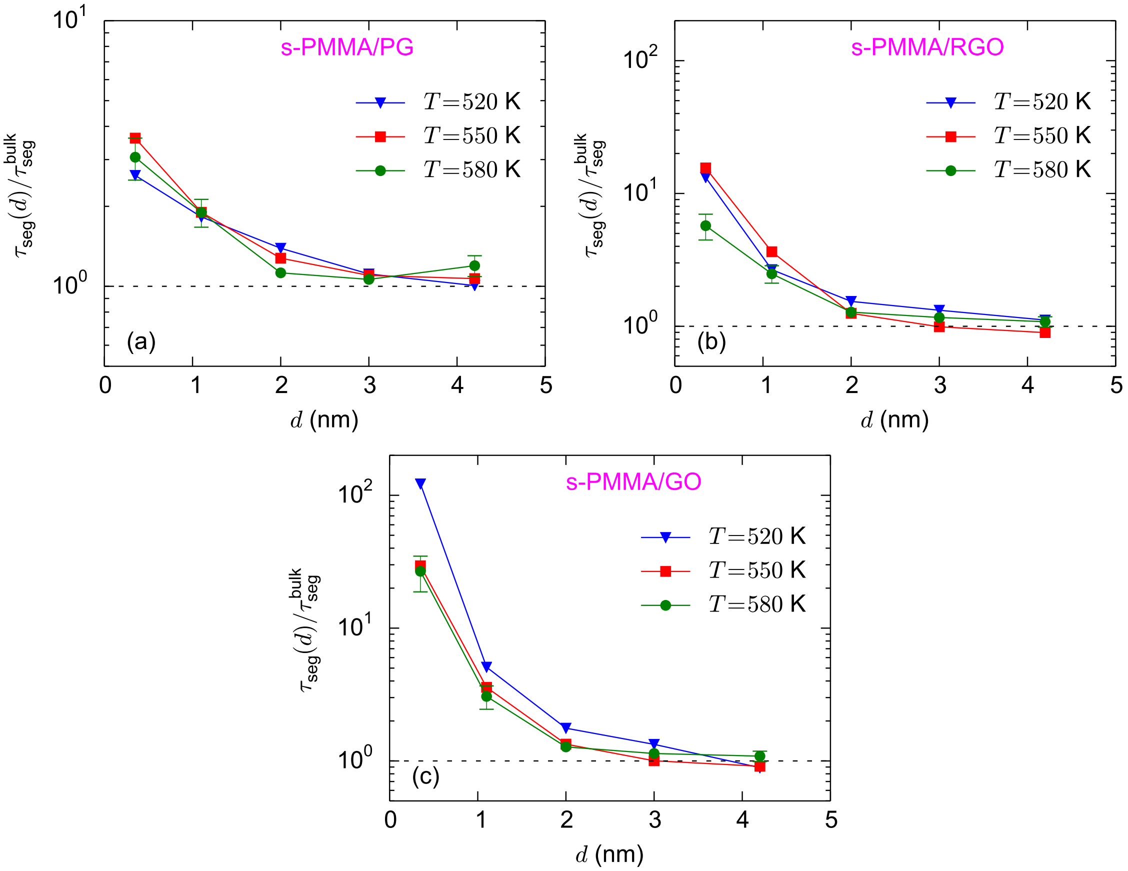


Figure S3: (a), (b), and (c) show the layer resolved values of the normalized relaxation times ($\frac{\tau_{\rm seg}(d)}{\tau_{\rm seg}^{\rm bulk}}$) for the s-PMMA/PG, s-PMMA/RGO, and s-PMMA/GO interfacial systems, at different temperatures, respectively. The error bars at $T = 580$ K have been estimated based on block averaging; at lower temperatures larger error bars are expected.
